# Fermentation Strategy and Non-*Saccharomyces* Yeast Strain Identity Jointly Shape Volatile Profiles in Chinese Steamed Bread Produced with Defined Composite Sourdoughs

**DOI:** 10.3390/foods15173064

**Published:** 2026-08-29

**Authors:** Xinyuan Hu, Yuxia Yang, Yue Li, Renyong Zhao, Shuangqi Tian, Zhanpeng Liu

**Affiliations:** 1 Henan Key Laboratory of Cereal Science and Technology, College of Food Science and Technology, Henan University of Technology, Zhengzhou 450001, China; 18637358365@163.com (X.H.); yangyuxia1999@163.com (Y.Y.); 15729397701@163.com (Y.L.); tianshuangqi@haut.edu.cn (S.T.); zhanpeng.liu@haut.edu.cn (Z.L.); 2 National Engineering Research Center for Wheat & Corn Further Processing, Zhengzhou 450001, China; 3 Food Laboratory of Zhongyuan, Luohe 462300, China

**Keywords:** Chinese steamed bread, composite sourdough, refrigerated sponge-dough, volatile organic compounds

## Abstract

Chinese steamed bread (CSB) is an important fermented wheat product, but controlled strategies for modulating its volatile profile remain limited. This study evaluated defined composite sourdoughs comprising *Wickerhamomyces anomalus* CRFSZY16 or *Kluyveromyces marxianus* FYY7 in combination with *Lactiplantibacillus plantarum* SDgsM3 for CSB production using the direct fermentation method (DFM) and the refrigerated sponge-dough method (RSDM). Technological stress tolerance, preliminary safety-related phenotypes, acidification capacity, volatile organic compounds (VOCs), multivariate profiles, and specific volume were assessed. SDgsM3 showed dough-relevant stress tolerance and rapid acidification, while both yeasts tolerated moderate fermentation stresses. Headspace solid-phase microextraction coupled with gas chromatography-triple quadrupole mass spectrometry (HS-SPME-GC-TQ-MS) revealed stage-dependent remodeling of volatile profiles from mature sourdough to fully proofed main dough and final CSB. RSDM selectively modified representative alcohols, aldehydes, furans, ketones, and esters rather than uniformly increasing all VOCs. In the final CSB, the *K. marxianus*-based RSDM system showed relatively higher levels of 3-methyl-1-butanol, whereas the *W. anomalus*-based RSDM system showed relatively higher levels of hexanal, nonanal, 1-hexanol, and 2-pentylfuran. Composite sourdough treatments also yielded specific volumes of 2.56–2.66 mL/g versus 2.44 mL/g for the commercial yeast control. These findings demonstrate strain- and process-dependent modulation of CSB volatile profiles and support flavor-oriented composite sourdough design.

## 1. Introduction

Chinese steamed bread (CSB), also known as mantou, is one of the most representative wheat-based staple foods in China and is widely consumed in both household and industrial settings [1,2]. Its quality is determined by multiple attributes, including volume, texture, crumb structure, appearance, staling behavior, and flavor perception [3]. Among these attributes, aroma is an important sensory factor affecting consumer acceptance, yet the volatile flavor profile of CSB has been less extensively characterized than that of baked bread products [4,5]. Commercial *Saccharomyces cerevisiae* (*S. cerevisiae*) is commonly used in modern steamed bread production because it provides rapid and reproducible leavening. However, products fermented solely with commercial *S. cerevisiae* often exhibit a relatively simple aroma profile compared with products fermented by traditional sourdoughs or mixed microbial starters [6,7]. Sourdough fermentation has therefore been considered a promising approach for improving the sensory complexity and technological quality of cereal products, including CSB [8]. Nevertheless, spontaneous or traditional sourdoughs may show unstable microbial composition and variable fermentation performance, which can limit their reproducibility in standardized production systems [9].

Sourdough is a complex cereal fermentation ecosystem shaped primarily by lactic acid bacteria (LAB) and yeasts, whose interactions influence acidification, leavening, substrate degradation, aroma formation, and product texture [10]. LAB contribute to cereal fermentation through the production of lactic acid, acetic acid, peptides, amino acid derivatives, exopolysaccharides, and other metabolites associated with flavor and structure development [9]. Yeasts contribute to dough expansion and aroma formation by producing carbon dioxide, ethanol, higher alcohols, aldehydes, esters, and other volatile metabolites [11]. A comprehensive review [12] of sourdough volatiles reported 196 volatile organic compounds (VOCs) in sourdough and sourdough bread, including aldehydes, alcohols, esters, ketones, acids, furans, pyrazines, lactones, sulfur compounds, and other compounds, illustrating the broad chemical basis of sourdough aroma. These VOCs may originate from carbohydrate metabolism, amino acid degradation, lipid oxidation, microbial esterification, and heat-induced reactions during steaming or baking. In CSB systems, Fu et al. [6] showed that sourdough microbial composition was associated with the texture and VOCs of the final product and that steamed breads prepared with different regional sourdoughs displayed distinct volatile profiles. Ran et al. [13] further demonstrated that mixed fermentation with sourdough and LAB increased hydrolyzed amino acids in dough and improved the specific volume, sensory quality, porosity, and storage stability of steamed buns. Collectively, these studies indicate that LAB-yeast sourdough fermentation can modulate both flavor development and technological quality formation in wheat-based steamed products, but its effects depend strongly on starter composition, fermentation conditions, and product matrix.

Non-*Saccharomyces* yeasts have received increasing attention in food fermentation because they can produce diverse secondary metabolites and may contribute sensory attributes that differ from those generated by conventional baker’s yeast. In bakery-related systems, non-*Saccharomyces* yeasts have been evaluated as adjunct cultures for improving aroma complexity, although their fermentation performance and flavor contribution are highly species-, strain-, and matrix-dependent. Zotta et al. [14] screened 32 wine-associated yeasts from Campania, Italy, and evaluated their decarboxylase activity, leavening ability, and volatile profiles in model doughs. The selected *Hanseniaspora uvarum* strains generated volatile profiles that differed from those produced by *S. cerevisiae*, particularly by reducing the proportion of aldehydes potentially associated with oxidative phenomena. Boscaino et al. [15] characterized 66 yeasts isolated from artisanal sourdoughs and mozzarella cheese whey and evaluated their physiological, technological, and aroma-related properties for bakery applications. When selected non-conventional yeasts were used in mixed inocula with *S. cerevisiae*, synergistic effects were observed, resulting in more complex dough volatile profiles as determined by solid-phase microextraction coupled with gas chromatography-mass spectrometry.

In CSB systems, Ding et al. [16] recently showed that co-fermentation of *Wickerhamomyces anomalus* with *S. cerevisiae* markedly reshaped volatile profiles during dough fermentation and steaming. In that study, 236 volatile compounds were detected across dough and steamed bread samples; alcohols were the most variable class, and a greater number of alcohols and esters showed increased relative abundance in the co-fermented dough than in the single *S. cerevisiae* dough. Phenylethyl alcohol increased from 0.59% in the unfermented control to 31.51% in *S. cerevisiae*-fermented dough and 35.29% in co-fermented dough; after steaming, its relative content increased to 39.12% and 46.01% in the corresponding steamed breads, respectively. Li et al. [17] investigated the combined use of *Torulaspora delbrueckii* Y22 and *S. cerevisiae* Y10 during dough fermentation for steamed bread production. The mixed culture improved gas-holding capacity and CO_2_ production in the dough, and a relatively high maltose concentration of 5.93 mg/g dry dough was observed after 12 h of fermentation. Moreover, co-fermentation enhanced the production of succinic acid, acetic acid, and essential amino acids in the fermented dough. Together, these studies support the use of selected non-*Saccharomyces* yeasts as functional adjuncts in wheat dough systems. However, most available evidence has focused on non-*Saccharomyces* yeasts co-fermented with *S. cerevisiae*, whereas the role of defined non-*Saccharomyces* yeast–LAB composite sourdoughs in the stage-dependent flavor evolution of CSB remains insufficiently understood.

Processing strategy is another critical determinant of volatile evolution because fermentation time, temperature, dough stage, and steaming can influence VOC formation, conversion, release, and retention [18]. The direct fermentation method (DFM) offers a relatively short pre-steaming transformation window. By contrast, the refrigerated sponge-dough method (RSDM) extends the pre-fermentation stage and can provide additional time for microbial metabolism and endogenous enzyme-mediated substrate conversion [19]. Wang et al. [20] reported that retarded sponge-dough processing improved the quality of Type I sourdough steamed bread and extended the time available for microbial metabolism and endogenous enzyme bioconversion, thereby contributing to product quality and flavor formation. However, prolonged fermentation or excessive acidification may also produce unfavorable effects, such as excessive sourness, altered gas retention, or changes in aroma balance, indicating that the relationship between sourdough fermentation intensity and product quality is not strictly linear. In addition, recent work continues to emphasize the importance of starter selection, microbial community structure, and processing conditions in shaping the flavor of steamed bread and other fermented cereal foods [21,22,23]. Consequently, two linked knowledge gaps remain: (i) how defined non-*Saccharomyces* yeast–*L. plantarum* consortia differ across successive stages from mature sourdough to final CSB and (ii) whether the same refrigerated sponge-dough strategy produces comparable VOC responses when the non-*Saccharomyces* strain is changed. Addressing these gaps is necessary for rational, strain-specific process design.

Therefore, this study prepared two defined composite sourdoughs by combining *Kluyveromyces marxianus* (*K. marxianus*) FYY7 or *Wickerhamomyces anomalus* (*W. anomalus*) CRFSZY16 with *Lactiplantibacillus plantarum* (*L. plantarum*) SDgsM3 and compared their effects on volatile profile evolution in CSB prepared by the direct fermentation method and the refrigerated sponge-dough method. Volatile organic compounds (VOCs) in mature sourdoughs, fully proofed main doughs, and CSB samples were analyzed using headspace solid-phase microextraction coupled with gas chromatography-triple quadrupole mass spectrometry (HS-SPME-GC-TQ-MS), and multivariate statistical analysis was used to compare volatile profiles and identify candidate discriminant VOCs. The main objective was to determine how non-*Saccharomyces* yeast–LAB composite sourdoughs and processing strategy jointly shape volatile profile development during CSB processing. This work provides a systematic basis for developing flavor-oriented composite sourdough technologies for CSB production.

## 2. Materials and Methods

### 2.1. Materials

Commercial wheat flour used for Chinese steamed bread preparation was obtained from Hebei Heima Flour Co., Ltd., Shijiazhuang, China. The basic compositional characteristics of the flour were as follows: moisture content, 14.07 ± 0.06%; protein content, 12.50 ± 0.05%; ash content, 0.47 ± 0.01%; and wet gluten content, 30.00 ± 0.14%. The flour was stored at 4 °C in sealed containers and equilibrated to room temperature before use.

Yeast extract-peptone-dextrose broth/agar (YPD), de Man, Rogosa and Sharpe broth/agar (MRS), yeast nitrogen base (YNB), glucose, fructose, maltose, sucrose, sodium chloride, ethanol, organic acids, antibiotics, and other analytical-grade reagents were purchased from Beijing Aoboxing Biotechnology Co., Ltd., Beijing, China. Sterile saline solution (0.85%, *w*/*v* NaCl) was used for cell washing and dilution. Unless otherwise stated, all media were sterilized by autoclaving at 121 °C for 15 min. Heat-labile carbohydrate solutions were sterilized by filtration through 0.22 μm membranes and added to autoclaved basal media after cooling to approximately 50 °C.

### 2.2. Microbial Strains and Culture Activation

Three food-derived strains previously isolated and purified from traditional Chinese fermented foods by our research group were used in this study. The two yeast strains were identified as *Wickerhamomyces anomalus* (*W. anomalus*) CRFSZY16 and *Kluyveromyces marxianus* (*K. marxianus*) FYY7, whereas the LAB strain was identified as *Lactiplantibacillus plantarum* (*L. plantarum*) SDgsM3. Species-level identification was performed in previous work by sequencing taxonomic marker genes and comparing the resulting sequences with those deposited in the NCBI database. The closest NCBI matches are listed in Supplementary Table S1. The strains were not selected through an exhaustive head-to-head screening of all available candidates. Rather, their selection was based on preliminary fermentation performance within our laboratory culture collection and on the expected complementary functions of the strains in the composite sourdough system. In preliminary wheat-based fermentation experiments, *W. anomalus* CRFSZY16 and *K. marxianus* FYY7 exhibited satisfactory fermentation performance and showed potential to enhance the aroma characteristics of fermented wheat products. This empirical selection was further supported by reported species-level metabolic characteristics related to aroma formation. *W. anomalus* has been reported to contribute to the formation of aroma-active volatile compounds, particularly esters, and to modify the volatile profile during CSB fermentation [16]. *K. marxianus* has been investigated as an alternative sourdough yeast because of its fermentation capacity and contribution to bread aroma volatiles [24]. *L. plantarum* SDgsM3 was included as the LAB component to provide acidification and complementary metabolic activity within the composite starter. In addition to its acidifying role, *L. plantarum* can substantially influence the volatile profile of sourdough; notably, *L. plantarum*-fermented sourdough has been reported to exhibit a characteristic volatile profile with relatively high levels of C4–C6 volatile compounds [25]. Thus, the three strains were selected to provide complementary fermentative and flavor-related functions in the composite sourdough system. Their strain-specific suitability for the present wheat-based system was subsequently evaluated experimentally through growth, stress tolerance, acidification, and preliminary safety-related assays, followed by assessment of their performance in the composite sourdough.

Before the experiments, yeast strains were activated twice in YPD broth at 30 °C for 18 h, while SDgsM3 was activated twice in MRS broth at 37 °C for 18 h. Activated cultures were harvested by centrifugation at 6000× *g* for 10 min, washed twice with sterile saline, and resuspended to the required cell density. Viable cell concentrations were confirmed by plate counting before inoculation.

### 2.3. Growth Kinetics and Evaluation of Technological Stress Tolerance

Growth kinetics of the three strains were determined by monitoring the optical density at 600 nm (OD600) during incubation under their respective optimal culture conditions. Yeast strains were cultivated in YPD broth at 30 °C, whereas SDgsM3 was cultivated in MRS broth at 37 °C. Activated cultures were inoculated at 1% (*v*/*v*), and the initial OD600 was adjusted to 0.06–0.08. OD600 was recorded at regular intervals during 24 h of incubation. Sterile uninoculated medium was used as the blank control.

Technological stress assays were selected to reflect the principal constraints expected for each microbial group rather than to provide a direct comparison using an identical stress panel across species. For *L. plantarum* SDgsM3, maltose concentration, low pH, and NaCl stress were emphasized because carbohydrate availability, acidification, and osmotic conditions are central to LAB performance in flour fermentation [26]. For the two yeasts, the test panel additionally included high glucose, ethanol, lactic acid, and acetic acid because yeast growth and fermentative activity can be affected by high sugar concentrations, accumulated ethanol, and organic acids in mixed fermentations [27]. After inoculation at 1% (*v*/*v*), SDgsM3 was incubated at 37 °C and the yeasts at 30 °C. Growth under each stress condition was assessed by OD_600_ after 24 h, with the corresponding unstressed culture condition used as the control. The resulting values were interpreted within each organism and were not used to rank LAB directly against yeasts.

### 2.4. Preliminary Safety Assessment of the Strains

A preliminary safety assessment was performed to evaluate the potential suitability of the strains for food fermentation applications [28,29]. For the two yeast strains, hemolytic activity, phospholipase activity, susceptibility to selected antimicrobial agents, and growth at 37 °C were evaluated. For SDgsM3, hemolytic activity, gelatinase activity, and antibiotic susceptibility were evaluated.

Antibiotic susceptibility of SDgsM3 was determined by the minimum inhibitory concentration (MIC) method. The tested antibiotics included ampicillin, vancomycin, gentamicin, kanamycin, erythromycin, clindamycin, tetracycline, and chloramphenicol. MIC values were determined and interpreted according to the relevant microbiological cut-off values for LAB. The susceptibility of the yeast strains to ampicillin (AMP, 10 μg/mL), chloramphenicol (Cm, 30 μg/mL), gentamicin (Gen, 10 μg/mL), tetracycline (TET, 30 μg/mL), erythromycin (E, 15 μg/mL), and streptomycin (S, 10 μg/mL) was evaluated via the disk diffusion assay.

### 2.5. Acidification Kinetics of L. plantarum SDgsM3

The acidification capacity of SDgsM3 was evaluated in MRS broth and in a liquid cereal-based fermentation medium (CBFM) to assess its performance under nutrient-rich and wheat-based conditions [30,31]. For the MRS system, SDgsM3 was inoculated into MRS broth at 1% (*v*/*v*) and incubated at 37 °C. For the CBFM, wheat flour and sterile water were mixed at 9:1 (*w*/*w*), and SDgsM3 was inoculated at 1% (*v*/*w*). Samples were collected at 0, 4, 8, 12, 16, 20, and 24 h, and pH was measured.

### 2.6. Preparation of Non-Saccharomyces Yeast–LAB Composite Sourdoughs

Two composite sourdoughs were prepared using the selected non-*Saccharomyces* yeasts and SDgsM3. The *W*-type composite sourdough was co-inoculated with *W. anomalus* CRFSZY16 and *L. plantarum* SDgsM3, whereas the *K*-type composite sourdough was co-inoculated with *K. marxianus* FYY7 and *L. plantarum* SDgsM3. The inoculation levels were adjusted to approximately 6.0 log CFU/g dough for the yeast strain and 7.0 log CFU/g dough for SDgsM3. Viable cell concentrations were confirmed by plate counting before inoculation.

To establish a firm sourdough model resembling Type I sourdough conditions, wheat flour and water were mixed at a flour-to-water ratio of 100:50 (*w*/*w*), corresponding to DY 150 [8]. The prepared doughs were fermented in sterile containers at 30 °C for 16 h. The resulting fermented doughs were regarded as mature sourdoughs (MSD) and were immediately used for subsequent CSB preparation.

### 2.7. Preparation of CSB

CSB formulations and ingredient allocations are provided in Supplementary Table S2. The formulation parameters, including the amount of composite sourdough incorporated into the dough, were determined and optimized through preliminary experiments conducted for the present study. The refrigerated sponge-dough method (RSDM), including the sponge preparation procedure and refrigerated fermentation conditions, was adopted from our previous studies [19,20] without modification. The commercial-yeast control contained 0.8% instant dry yeast on a flour-weight basis and was included as a conventional process benchmark rather than an inoculum-matched microbiological control. Because the commercial-yeast control and the composite sourdough treatments differed in starter composition, inoculum characteristics, and fermentation history, comparisons with the control were interpreted as overall starter- or process-level comparisons rather than as estimates of the isolated effect of any single microorganism.

For the direct fermentation method (DFM), the mature composite sourdough was incorporated directly into the final-dough formulation according to Supplementary Table S2, after which the dough proceeded to the common mixing, dividing, shaping, proofing, and steaming sequence. For the refrigerated sponge-dough method (RSDM), processing was conducted in two stages. First, a sponge was prepared according to the ingredient allocation specified in Supplementary Table S2 and held under refrigerated conditions at 4–8 °C for 24 h. After refrigerated holding, the sponge was combined with the remaining final-dough ingredients, and the resulting dough was subjected to the same subsequent processing sequence as the DFM treatment. Thus, the principal difference between DFM and RSDM was the inclusion of a 24 h refrigerated sponge stage at 4–8 °C before final dough formation in RSDM. After final ingredient incorporation, all treatments were mixed at low speed for 3 min and high speed for 3 min, divided into 75 g portions, sheeted and shaped, proofed at 38 °C and 85% relative humidity for 40 min, steamed for 20 min under identical operating conditions, and cooled at room temperature for 1 h before analysis.

### 2.8. Determination of Specific Volume

After cooling to ambient temperature, CSB samples were placed on the platform of a laser volume analyzer. The height, width/diameter, and volume of each sample were measured according to the manufacturer’s guidelines to calculate the specific volume (mL/g). All measurements were performed using five independent biological fermentation batches.

### 2.9. Determination of VOCs by HS-SPME-GC-TQ-MS

VOCs were determined in samples representing four process stages: mature sourdough (MSD), mature refrigerated sponge dough (MRSD; RSDM treatments only), fully proofed main dough (MD), and Chinese steamed bread (CSB) crumb. Headspace solid-phase microextraction coupled with gas chromatography-triple quadrupole mass spectrometry (HS-SPME-GC-TQ-MS) was performed in full-scan acquisition mode. The method was adapted from published volatile-analysis protocols for cereal-based fermented products [5,32,33,34], with modifications to extraction conditions and chromatographic parameters to suit dough and CSB matrices.

Methyl heptanoate was used as the internal standard. A stock solution of methyl heptanoate was prepared in chromatographic-grade anhydrous ethanol at 1000 μg/mL and stored at −20 °C in the dark. Before analysis, the stock solution was diluted with ultrapure water to obtain a 10 μg/mL working solution. For each analysis, 2.00 g of dough or CSB crumb sample was accurately weighed into a 20 mL headspace vial. Then, 5.00 mL of 20% (*w*/*v*) NaCl solution and 5 μL of methyl heptanoate working solution were added. The vial was immediately sealed with a polytetrafluoroethylene (PTFE)/silicone septum cap and equilibrated at 50 °C for 10 min. A preconditioned 50/30 μm divinylbenzene/carboxen/polydimethylsiloxane (DVB/CAR/PDMS) SPME fiber was conditioned at 250 °C for 20 min before extraction. The fiber was exposed to the vial headspace at 50 °C for 40 min under continuous agitation, followed by thermal desorption in the GC injection port at 250 °C for 5 min.

Chromatographic separation was carried out on a DB-Wax polar capillary column (30 m × 0.25 mm × 0.25 μm). High-purity helium was used as the carrier gas at a constant flow rate of 1.0 mL/min. The injector temperature was maintained at 250 °C, and injection was performed with a split ratio of 1:1. The oven program was as follows: 40 °C for 2 min, increased to 50 °C at 2 °C/min, increased to 110 °C at 5 °C/min, then increased to 230 °C at 3 °C/min and held for 7 min. Mass spectrometric detection was performed using electron-impact ionization at 70 eV. The ion source, transfer line, and quadrupole temperatures were set at 230 °C, 280 °C, and 150 °C, respectively. Data were acquired in full-scan mode over *m*/*z* 35–350. A C7–C30 n-alkane mixture was analyzed separately under the same chromatographic conditions to calculate retention index (RI). The alkane standard mixture was not added to the sample vials.

VOCs were tentatively identified by comparing mass spectra with National Institute of Standards and Technology (NIST) spectral libraries and by matching experimentally determined RI values with literature RI values reported for comparable polar columns. Peaks with substantial blank interference, poor chromatographic resolution, low spectral similarity, or large RI deviation were excluded. Only compounds with a spectral match factor ≥80 and an RI deviation within ±20 were retained for subsequent screening. Because identification was based on spectral-library matching and retention indices, the reported compound identities were treated as tentative MS/RI assignments. Only compounds detected in at least three of five biological replicates in at least one treatment and showing no substantial procedural-blank interference were retained in the final VOC matrix. Relative abundance was calculated as the peak-area ratio of each target VOC to methyl heptanoate, the internal standard; these normalized values are not absolute concentrations.

Odor descriptors for retained VOCs were compiled from published aroma studies on CSB, sourdough, bread, and other cereal-based fermented foods whenever available. For compounds not clearly described in closely related matrices, descriptors were supplemented from established flavor databases, including The Good Scents Company, FlavorDB/FlavorDB2, and Flavornet. When multiple descriptors existed, commonly reported food-relevant terms were standardized. These annotations were used only as literature-based contextual information: they do not constitute sensory measurements of the present CSB samples and should not be interpreted as evidence that a corresponding odor note was perceived. Compounds suspected to originate from environmental background, plastic materials, solvents, or analytical artifacts were not considered candidate aroma-relevant VOCs.

### 2.10. Multivariate Statistical Analysis and Screening of Candidate Discriminant VOCs

All VOC data matrices were checked prior to multivariate statistical analysis and subjected to Pareto scaling to reduce the influence of variables with high relative abundance while retaining the contribution of variables with intermediate abundance. Principal component analysis (PCA) was performed as an unsupervised approach to visualize the overall variation and distribution patterns of VOC profiles among treatment groups.

Orthogonal partial least squares-discriminant analysis (OPLS-DA) was performed using SIMCA-P version 14.1 to further characterize treatment-associated differences in VOC profiles and to identify variables contributing to group separation. Model performance was evaluated using R^2^X(cum), R^2^Y(cum), and Q^2^(cum). Internal model validation was conducted by seven-fold cross-validation, and model robustness and potential overfitting were further assessed using 200 Y-permutation tests. The R^2^ and Q^2^ intercepts obtained from the permutation tests were considered together with the model parameters when evaluating model validity [35,36].

Candidate discriminant VOCs were screened using the combined criteria of variable importance in projection (VIP) > 1.0 and *p* < 0.05 [37]. The VIP values and corresponding *p* values for individual VOCs were obtained from the SIMCA-P analysis, and only compounds satisfying both criteria were considered candidate discriminant VOCs. For pairwise OPLS-DA comparisons, S-plots were additionally used to visualize the covariance and correlation of individual VOCs with the predictive component. Candidate discriminant VOCs were interpreted in conjunction with their relative-abundance patterns and the validation performance of the corresponding OPLS-DA models [38].

### 2.11. Statistical Analysis

Multivariate statistical analyses were conducted using SIMCA-P software version 14.1. Differences in specific volume among CSB treatment groups were evaluated by one-way analysis of variance (ANOVA), followed by Duncan’s multiple range test for post hoc comparisons. Differences were considered statistically significant at *p* < 0.05. Pearson correlation analysis was used to evaluate linear associations among VOC relative abundances. The statistical significance of Pearson correlation coefficients was denoted as * *p* < 0.05, ** *p* < 0.01, and *** *p* < 0.001. Data visualization was performed using Origin 2024. Unless otherwise specified, all experiments were carried out with at least three biological replicates.

For VOC and specific-volume analyses, five independent biological fermentation batches were used. Each biological replicate represented an independently prepared and inoculated fermentation batch that underwent the complete fermentation, processing, steaming, sampling, and analytical procedures. Samples collected from different stages within the same batch were treated as repeated measurements and were not considered independent biological replicates. To facilitate understanding of the experimental design, a schematic diagram is provided in Supplementary Figure S1, illustrating the two yeast–LAB composite consortia, the commercial yeast control, the two fermentation strategies, the four sampling stages, and the number of independent biological replicates.

## 3. Results

### 3.1. Growth Characteristics and Technological Stress Tolerance of the Starter Strains

**Figure 1:**
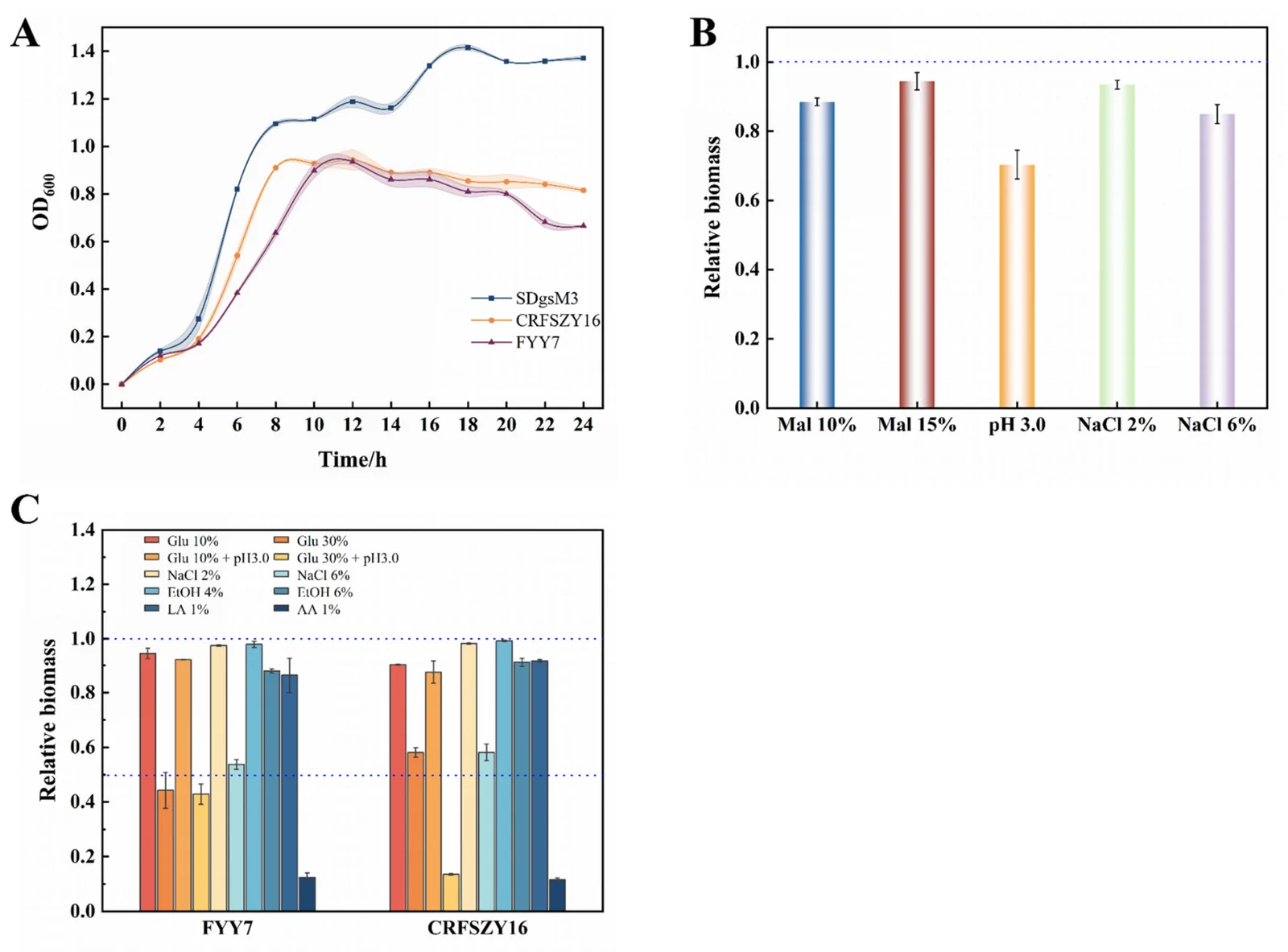
Growth characteristics and technological stress tolerance of the starter strains. (**A**) Growth kinetics of *L. plantarum* SDgsM3, *W. anomalus* CRFSZY16, and *K. marxianus* FYY7. (**B**) Relative biomass of *L. plantarum* SDgsM3 under maltose, NaCl, and acidic stress conditions. (**C**) Relative biomass of *W. anomalus* CRFSZY16 and *K. marxianus* FYY7 under glucose, combined glucose and acid stress, NaCl, ethanol, lactic acid, and acetic acid stress conditions. The dotted horizontal line at a relative biomass of 1.0 in panels B and C represents the normalized level of the corresponding unstressed control. In panel C, the additional dotted horizontal line at 0.5 indicates the 50% relative-biomass reference level.

All three strains showed a short adaptation period followed by active growth under their respective culture conditions (Figure 1A). *L. plantarum* SDgsM3 entered the exponential phase after approximately 4 h, showed a temporary slowdown after 8 h, and reached its highest growth level at approximately 18 h. Its biomass then remained relatively stable until 24 h, indicating strong growth capacity and good late-stage stability. The two non-*Saccharomyces* yeasts also grew rapidly after 4 h. CRFSZY16 reached a relatively high growth level at approximately 8–12 h and subsequently declined slowly, whereas FYY7 reached its maximum at approximately 10–12 h and showed a more pronounced decline after 20 h.

Within the LAB-specific stress panel (Figure 1B), SDgsM3 maintained relatively high biomass at 10% and 15% maltose and under 2% NaCl, while 6% NaCl produced a moderate reduction. pH 3.0 caused the strongest inhibition in this panel, lowering relative biomass to approximately 0.70 without completely suppressing growth. These data indicate that SDgsM3 retained growth across several stress levels relevant to flour fermentation, although the assays do not by themselves predict performance in dough. As shown in Figure 1C, FYY7 and CRFSZY16 retained relatively high biomass under 10% glucose, 10% glucose plus pH 3.0, 2% NaCl, 4–6% ethanol, and 1% lactic acid. Both strains were more strongly inhibited by 30% glucose, 6% NaCl, and particularly acetic acid. CRFSZY16 showed slightly higher relative biomass than FYY7 under 30% glucose alone, whereas FYY7 retained more biomass under the combined 30% glucose and pH 3.0 condition. These results show strain-dependent responses to high osmotic pressure and combined stress and identify acetic acid as the most inhibitory condition among those tested.

Overall, SDgsM3, FYY7, and CRFSZY16 retained growth under several fermentation-relevant stress conditions, supporting their technological suitability for further evaluation in the sourdough system.

### 3.2. Acidification Capacity of L. plantarum

**Figure 2:**
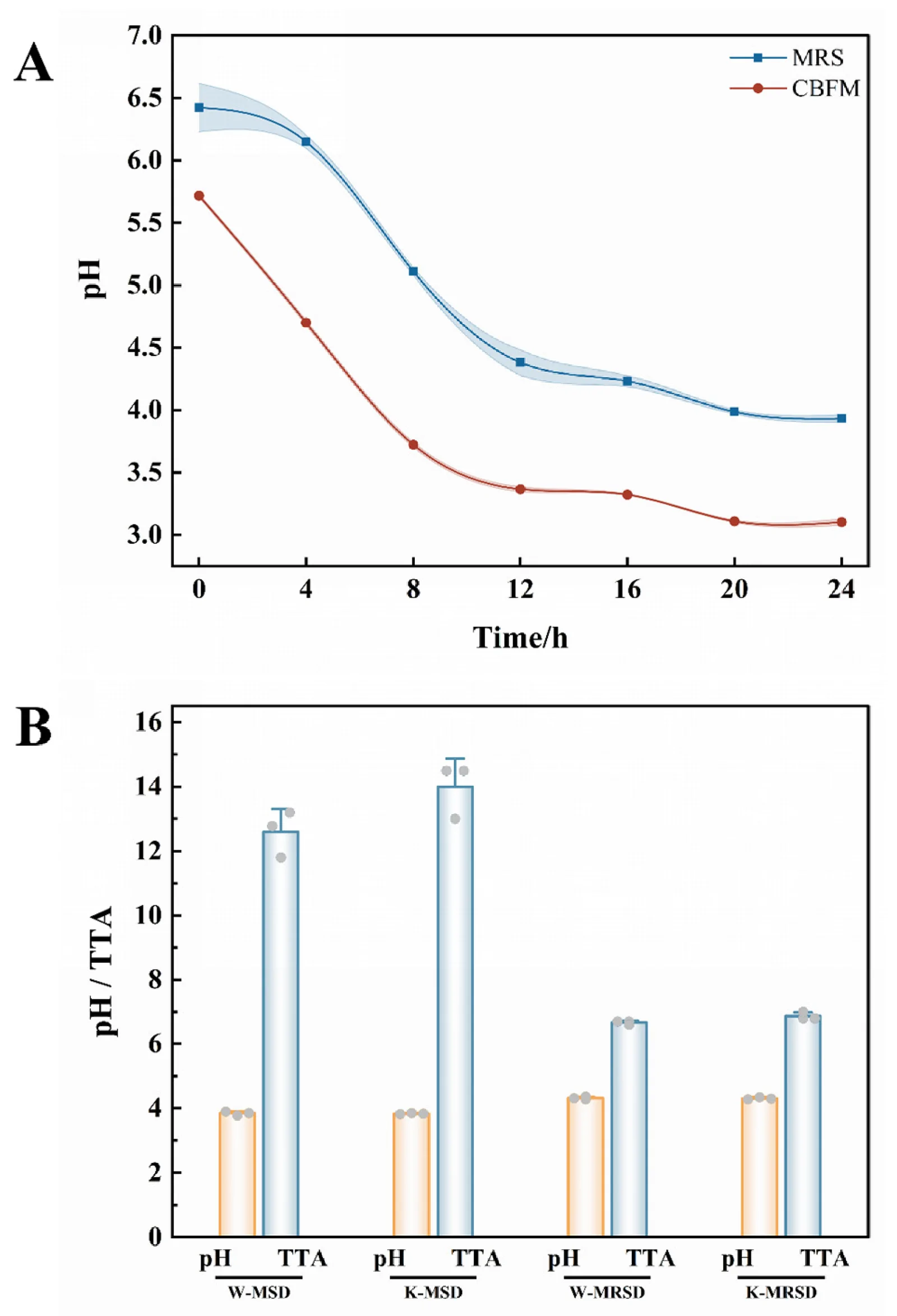
Acidification characteristics of *L. plantarum* SDgsM3 and the composite dough systems. (**A**) Acidification kinetics of *L. plantarum* SDgsM3 in MRS broth and the CBFM. (**B**) Endpoint pH and TTA of *W*-MSD, *K*-MSD, *W*-MRSD, and *K*-MRSD. Data are presented as mean ± SD. In panel A, the shaded areas represent the standard deviation around the mean. In panel B, the individual dots represent independent biological replicates.

Figure 2A shows that *L. plantarum* SDgsM3 exhibited clear acidification capacity in both MRS broth and the CBFM system, although the acidification kinetics differed between the two matrices. In MRS broth, the initial pH was approximately 6.4. The pH decreased slowly during the first 4 h, followed by a rapid decline between 4 and 12 h. After 12 h, the acidification rate gradually slowed, and the pH tended to stabilize between 20 and 24 h, reaching a final value of approximately 3.9. By contrast, SDgsM3 showed a faster apparent pH decrease in the CBFM system. The pH decreased rapidly from an initial value of approximately 5.7 to about 3.7 within 8 h, and further declined to approximately 3.4 at around 12 h. Thereafter, the pH entered a relatively stable phase and finally remained at approximately 3.1. These results indicate that SDgsM3 was able to acidify both media and adapted well to the wheat-flour matrix.

MRS broth and the CBFM system differ in initial pH, nutrient composition, and buffering capacity. Therefore, the extent of pH reduction in the two systems cannot be interpreted directly as equivalent total acid production. More precisely, the results demonstrate acidification ability in both systems, with a faster apparent pH decrease in the flour-based model.

### 3.3. Preliminary Safety Assessment of the Starter Strains

No hemolysis was detected for the three strains under the assay conditions. The two yeasts were negative for the tested phospholipase phenotype, and SDgsM3 was negative for gelatinase activity. The antimicrobial susceptibility results did not reveal an obvious acquired-resistance concern within the limited panel tested. These findings support continued evaluation of the strains as food-fermentation starters, but they represent a preliminary phenotypic screen rather than a comprehensive food-safety assessment.

### 3.4. Endpoint Viable Counts and Acidification Characteristics of the Composite Sourdough Systems

At the fermentation endpoint, all four composite dough systems maintained high viable counts: *L. plantarum* SDgsM3 reached approximately 9.0 log CFU/g and the yeasts approximately 8.0 log CFU/g. Endpoint acidification differed numerically among conditions (Figure 2B). The *W*- and *K*-based mature sourdoughs had final pH values of approximately 3.8 and mean TTA values of approximately 12.7 and 14.0 mL, respectively, whereas the corresponding refrigerated sponge doughs had pH values of approximately 4.3 and TTA values of approximately 6.7 and 6.9 mL. The numerical differences between process stages were therefore larger than those between the *W*- and *K*-based starters within the same stage.

### 3.5. Volatile Profiles of Mature Composite Sourdoughs and Mature Refrigerated Sponge Doughs

**Figure 3:**
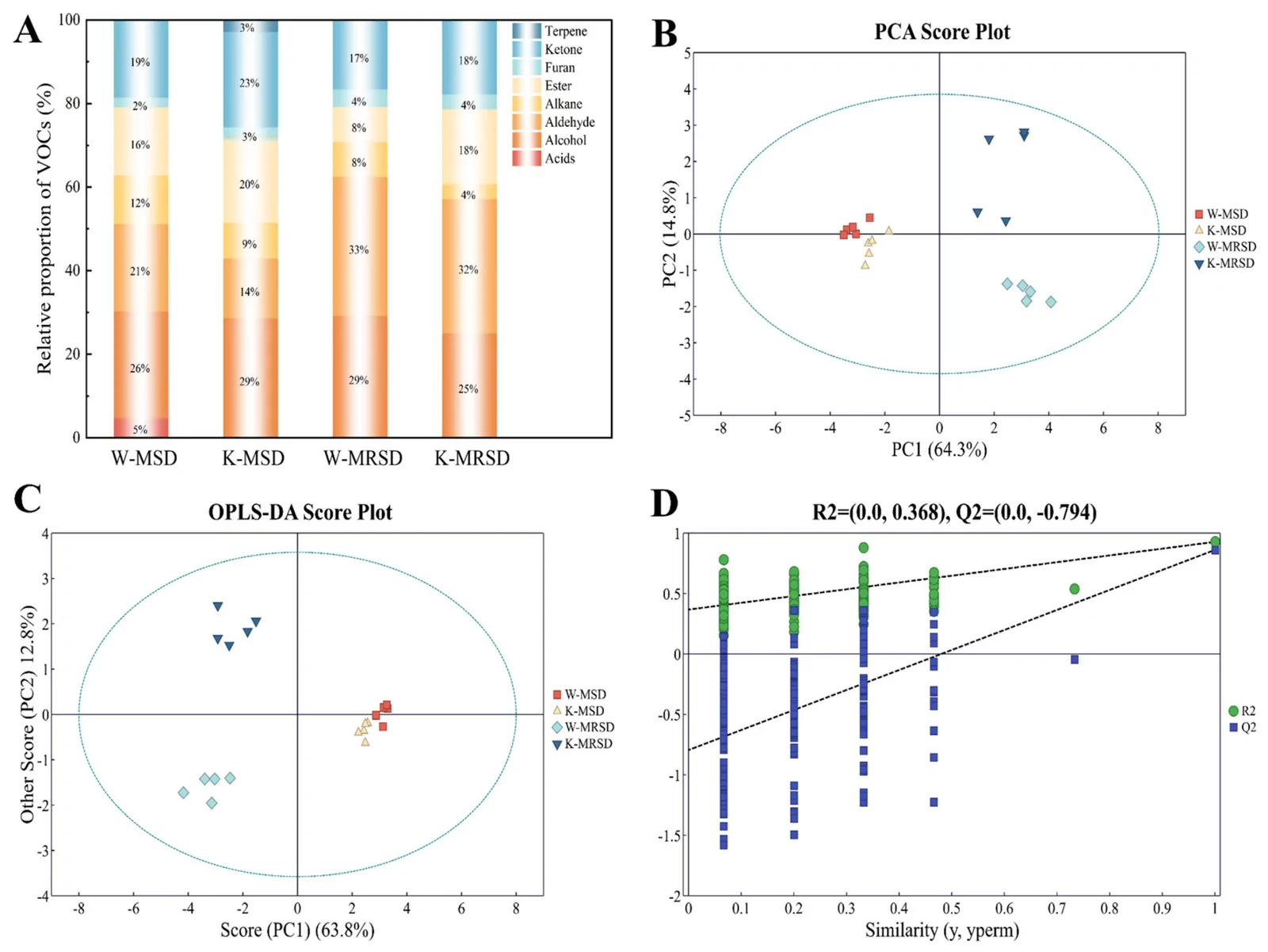
Volatile composition and multivariate analysis of mature composite sourdoughs (MSD) and mature refrigerated sponge doughs (MRSD). (**A**) Relative distribution of volatile compound classes in *W*-MSD, *K*-MSD, *W*-MRSD, and *K*-MRSD. (**B**) PCA score plot. (**C**) Exploratory OPLS-DA score plot. (**D**) Permutation test of the OPLS-DA model. The ellipses in panels B and C indicate the 95% confidence regions. In panel D, the dashed lines represent the regression lines of R^2^ and Q^2^ values obtained from the permutation test.

To compare VOC profiles between mature composite sourdoughs and mature refrigerated sponge doughs, VOC composition, relative abundance (Supplementary Table S3), and multivariate characteristics (Figure 3) were analyzed in *W*-MSD, *K*-MSD, *W*-MRSD, and *K*-MRSD. The mean summed internal-standard-normalized VOC signals of the four groups were 10.051, 13.766, 22.614, and 22.226, respectively. Compared with the mature composite sourdoughs, the mature refrigerated sponge doughs showed higher summed internal-standard-normalized VOC signals. Specifically, the summed internal-standard-normalized VOC signal increased approximately 2.25-fold from *W*-MSD to *W*-MRSD and approximately 1.61-fold from *K*-MSD to *K*-MRSD, indicating a larger relative increase in the *W*-based system. Regarding volatile-class composition (Figure 3A), alcohols, aldehydes, esters, and ketones were the major volatile classes detected in all four groups, although their proportional distributions differed among treatments. In *W*-MSD, alcohols and aldehydes accounted for 26% and 21% of the summed internal-standard-normalized VOC signal, respectively. *K*-MSD showed a broadly similar class profile but relatively higher proportions of alcohols, esters, and ketones and a lower proportion of aldehydes. After refrigerated sponge-dough processing, the proportion of aldehydes increased to 33% and 32% of the summed internal-standard-normalized VOC signal in *W*-MRSD and *K*-MRSD, respectively. The ester proportion decreased markedly in *W*-MRSD compared with *W*-MSD, whereas *K*-MRSD retained a relatively high ester proportion. These patterns indicate selective remodeling of the volatile profile rather than uniform amplification of all volatile classes.

PCA showed an unsupervised shift from the mature sourdoughs toward the refrigerated sponge doughs (Figure 3B), with PC1 and PC2 explaining 64.3% and 14.8% of the variance. The exploratory OPLS-DA model (Figure 3C) yielded R^2^X(cum) = 0.936, R^2^Y(cum) = 0.950, and Q^2^(cum) = 0.895; its permutation test produced a negative Q^2^ intercept (Figure 3D). Taken together, these metrics support treatment-related structure in the VOC matrix, although the supervised separation should be interpreted as exploratory rather than as independent evidence of a biological mechanism.

**Figure 4:**
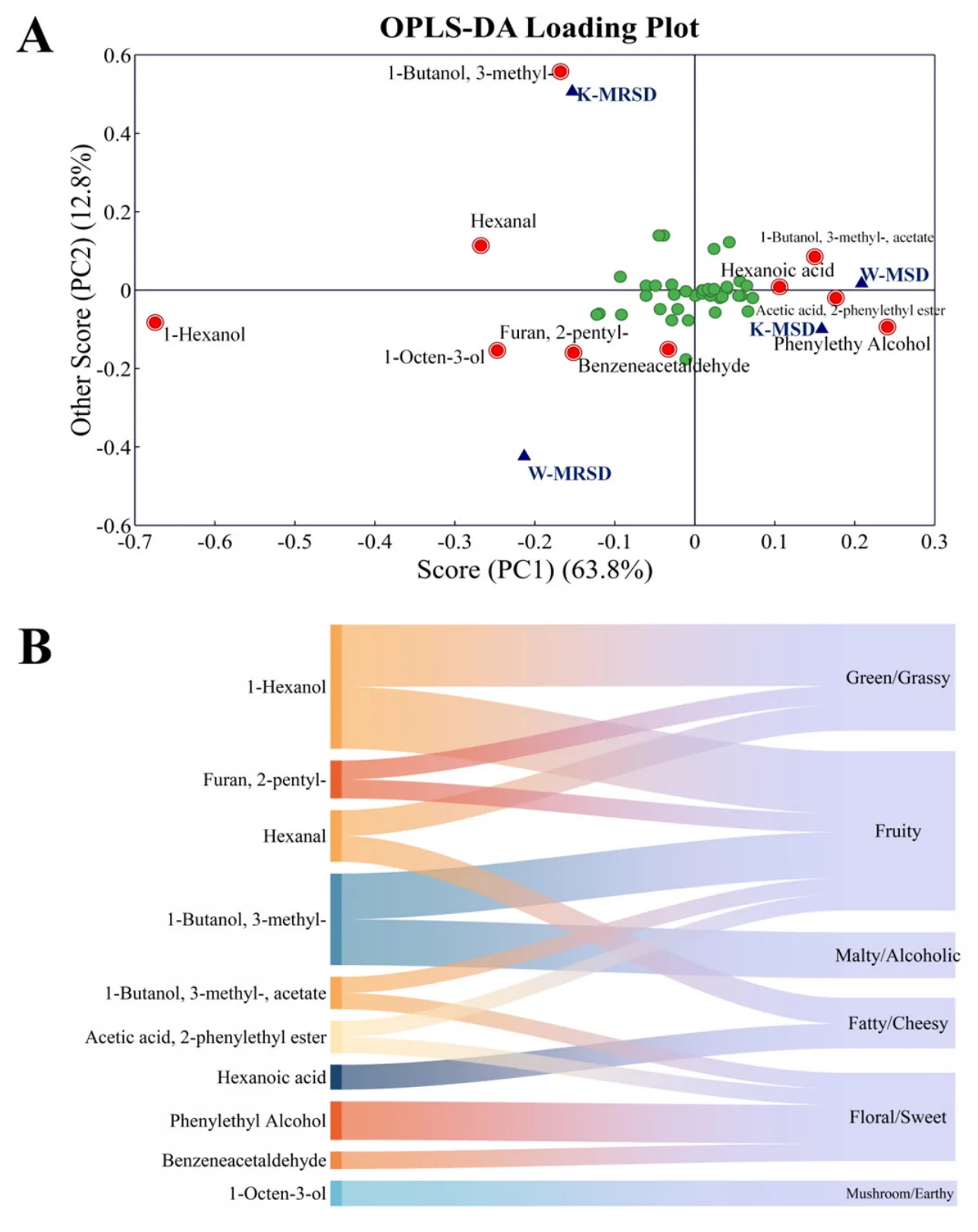
Candidate discriminant volatile compounds and their literature-reported odor descriptors in mature composite sourdoughs (MSD) and mature refrigerated sponge doughs (MRSD). (**A**) OPLS-DA loading plot showing the contribution of individual VOCs to sample discrimination; compounds with variable importance in projection (VIP) values > 1 and *p* < 0.05 were considered candidate discriminant VOCs. Red circles indicate candidate discriminant VOCs, green circles indicate the remaining VOC variables, and blue triangles indicate the treatment groups. (**B**) Sankey diagram linking candidate discriminant VOCs to odor descriptors reported in the literature, with line width proportional to the corresponding VIP value. The odor descriptors were obtained from published literature and were not experimentally determined in the present study.

In the OPLS-DA loading plot (Figure 4A), variables with VIP > 1 were treated as candidate discriminant VOCs rather than as independently validated biomarkers. Phenylethyl alcohol and 2-phenylethyl acetate showed higher relative abundances in the mature sourdoughs and were markedly reduced or undetected after the refrigerated sponge stage. Conversely, 1-hexanol increased from 2.660 and 4.588 in *W*-MSD and *K*-MSD to 13.196 and 11.046 in *W*-MRSD and *K*-MRSD, respectively, while hexanal increased from 0.066 and 0.198 to 1.470 and 1.744. 2-Pentylfuran and 1-octen-3-ol also showed higher relative abundances in the mature refrigerated sponge doughs, particularly in the *W*-based system. The two MRSD groups also differed in their candidate discriminant VOC patterns. 3-Methyl-1-butanol was most prominent in *K*-MRSD (mean relative abundance, 3.270), whereas *W*-MRSD showed relatively higher abundances of 1-hexanol, 2-pentylfuran, 1-octen-3-ol, and benzeneacetaldehyde; 1-octen-3-ol reached 2.346 and benzeneacetaldehyde 0.476 in *W*-MRSD. These differences indicate that the net VOC outcome of the refrigerated sponge stage depended on the non-*Saccharomyces* strain used with the common LAB partner. Figure 4B links the candidate discriminant VOCs to odor descriptors reported in the literature. The width of each connecting line represents the VIP value of the corresponding compound and therefore its contribution to group discrimination in the OPLS-DA model. According to the literature annotations, 1-hexanol and hexanal were mainly associated with green/grassy descriptors; 2-pentylfuran, 3-methylbutyl acetate, and some aldehydes with fruity descriptors; 3-methyl-1-butanol with malty/alcoholic descriptors; hexanoic acid with fatty/cheesy descriptors; phenylethyl alcohol, benzeneacetaldehyde, and 2-phenylethyl acetate with floral/sweet descriptors; and 1-octen-3-ol with mushroom/earthy descriptors. These annotations are provided only to contextualize the chemical identities. Because sensory evaluation, GC-olfactometry, and odor-activity-value determination were not performed, the actual perceived aroma characteristics of the samples cannot be inferred from these descriptors.

Overall, the refrigerated sponge stage was associated with a higher summed internal-standard-normalized VOC signal and a shift toward larger contributions from aldehydes, selected alcohols, and furans. The specific compounds driving that shift differed between the *W*- and *K*-based systems.

### 3.6. Volatile Profiles of Fully Proofed Main Doughs Prepared with Different Starters

**Figure 5:**
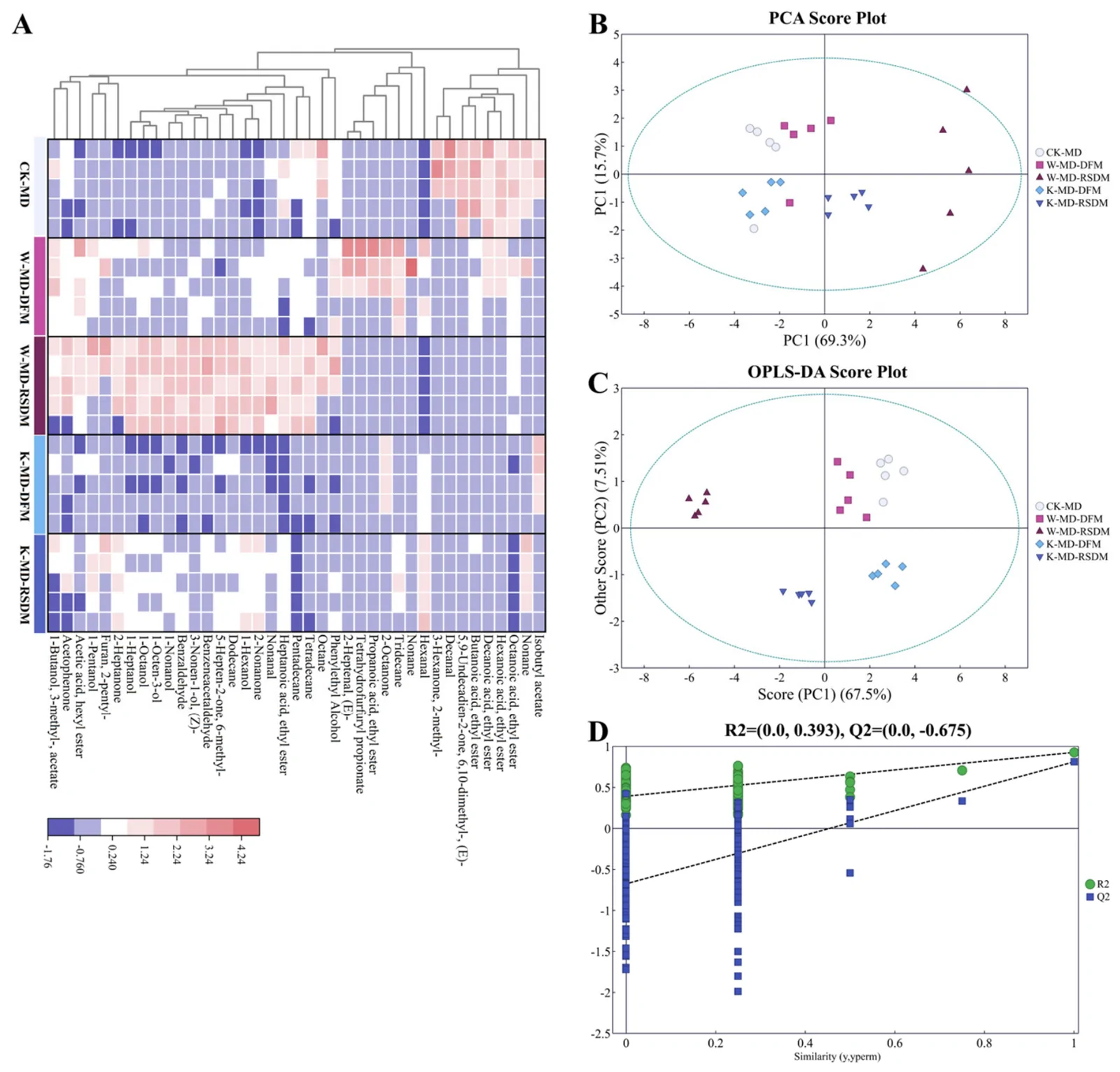
Volatile compound distribution and multivariate analysis of fully proofed main doughs (MD). (**A**) Heatmap of relative VOC abundance in the five dough groups. (**B**) PCA score plot. (**C**) Exploratory OPLS-DA score plot. (**D**) Permutation test of the OPLS-DA model. The ellipses in panels B and C indicate the 95% confidence regions. In panel D, the dashed lines represent the regression lines of R^2^ and Q^2^ values obtained from the permutation test.

To characterize the influence of different starter systems on the volatile profiles of fully proofed main doughs, VOC composition, relative abundance (Supplementary Table S4), and multivariate distribution patterns (Figure 5) were compared among CK-MD, *W*-MD-DFM, *W*-MD-RSDM, *K*-MD-DFM, and *K*-MD-RSDM. *W*-MD-RSDM exhibited the highest summed internal-standard-normalized VOC signal, followed by *K*-MD-RSDM and *W*-MD-DFM, whereas *K*-MD-DFM had a lower value than CK-MD. Relative to CK-MD, the summed internal-standard-normalized VOC signal increased by approximately 41.0% in *W*-MD-DFM, 169.1% in *W*-MD-RSDM, and 64.8% in *K*-MD-RSDM, but decreased by approximately 14.8% in *K*-MD-DFM. The heatmap further revealed distinct distributions of individual VOCs among the five fully proofed main doughs (Figure 5A). CK-MD was characterized by relatively higher levels of several ethyl esters, including octanoic acid, ethyl ester (0.891) and hexanoic acid, ethyl ester (0.442). In contrast, *W*-MD-DFM and *W*-MD-RSDM showed higher relative abundances of several alcohols, aldehydes, and ketones, particularly 1-hexanol, 1-heptanol, 1-octen-3-ol, benzaldehyde, benzeneacetaldehyde, 2-heptanone, and 2-nonanone. *K*-MD-RSDM also showed increases in selected alcohols and ketones, although the overall intensity was lower than that observed in *W*-MD-RSDM. By comparison, *K*-MD-DFM showed relatively low abundances of multiple VOCs, yielding a volatile profile distinct from those of the other composite sourdough main doughs. Overall, the changes at the fully proofed dough stage were compound- and treatment-specific.

PCA showed a clear separation tendency among the overall VOC profiles of the five fully proofed main doughs (Figure 5B). PC1 and PC2 explained 69.3% and 15.7% of the total variance, respectively, with a cumulative contribution of 85.0%. The five treatment groups occupied different regions of the PCA score space, indicating treatment-associated variation in their overall VOC profiles. *W*-MD-RSDM was most clearly separated from the other groups, consistent with its relatively high summed internal-standard-normalized VOC signal and the enrichment of several alcohols, aldehydes, and ketones. OPLS-DA further characterized the multivariate differences among the five groups (Figure 5C). The permutation test yielded a negative Q^2^ intercept of −0.675 (Figure 5D), providing additional support for the observed treatment-associated separation.

The fully proofed dough data thus show that both starter identity and process history were associated with the net VOC pattern before steaming. In particular, the *W*-based RSDM dough combined the highest summed internal-standard-normalized VOC signal with enrichment of several alcohols, aldehydes, and ketones, whereas the *K*-based DFM dough showed comparatively low levels of several esters and aromatic aldehydes.

### 3.7. Volatile Profiles of CSB Prepared with Composite Sourdoughs

**Figure 6:**
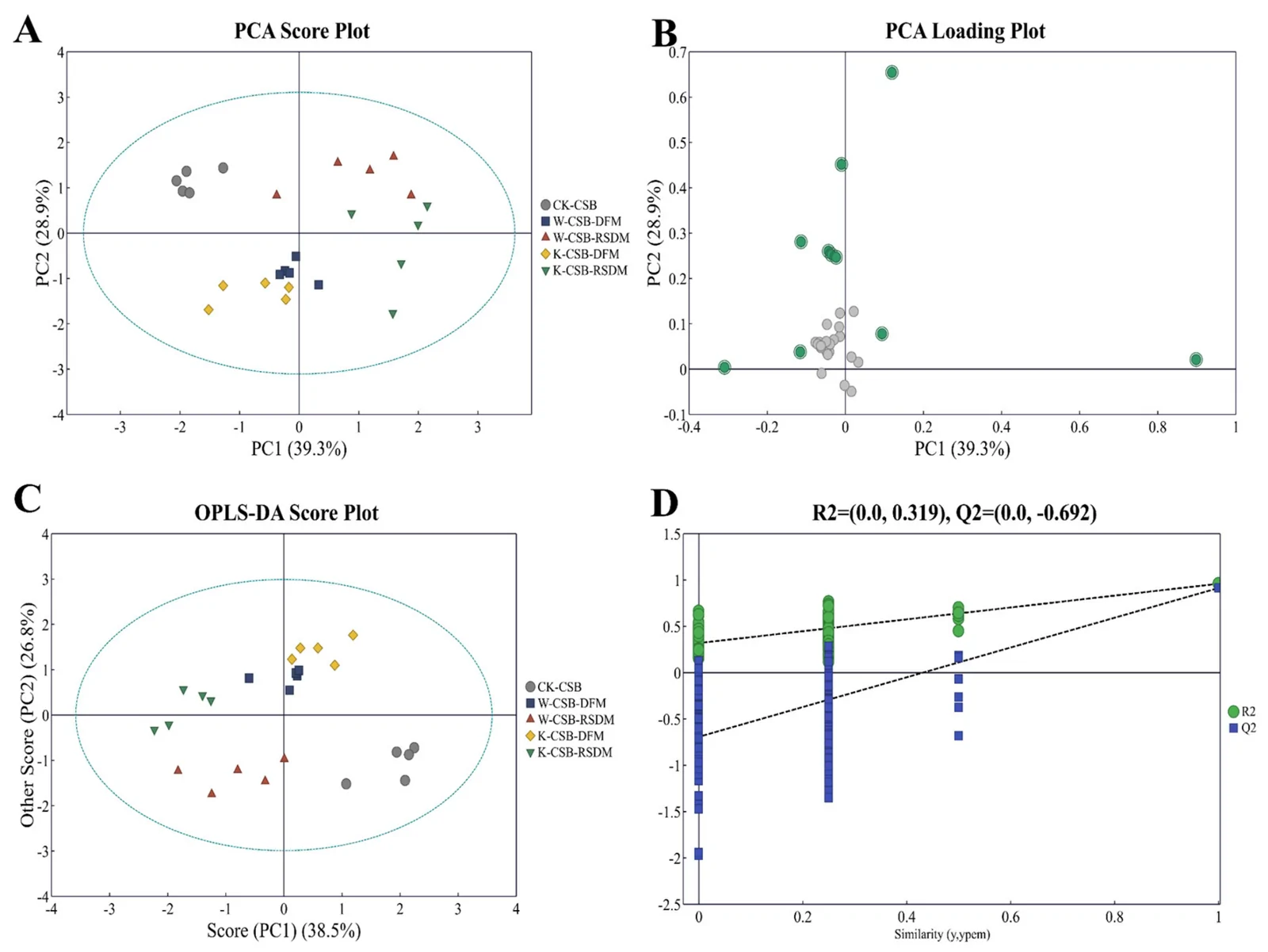
Multivariate analysis of volatile profiles in Chinese steamed bread (CSB). (**A**) PCA score plot of the five CSB groups. (**B**) PCA loading plot showing the contributions of individual VOCs to sample separation. Green dots indicate the highlighted VOCs, whereas gray dots indicate the remaining VOC variables. (**C**) Exploratory OPLS-DA score plot. (**D**) OPLS-DA permutation test. The ellipses in panels A and C indicate the 95% confidence regions. In panel D, the dashed lines represent the regression lines of R^2^ and Q^2^ values obtained from the permutation test.

To evaluate the effects of composite sourdough starters on the VOC profiles of CSB, VOC composition (Supplementary Table S5) and multivariate distribution patterns (Figure 6) were compared among CK-CSB, *W*-CSB-DFM, *W*-CSB-RSDM, *K*-CSB-DFM, and *K*-CSB-RSDM. PCA showed a clear separation tendency among the five CSB groups (Figure 6A). PC1 and PC2 explained 39.3% and 28.9% of the total variance, respectively, with a cumulative contribution of 68.2%. The five groups occupied different regions of the PCA score space, indicating treatment-associated variation in the final CSB volatile profiles. The PCA loading plot (Figure 6B) showed that separation was driven by a limited subset of VOCs with relatively high loading values rather than by uniform changes across all detected compounds. OPLS-DA further characterized these multivariate differences (Figure 6C). The five groups occupied distinct regions in the score space. In addition, the permutation test produced a negative Q^2^ intercept of −0.692 (Figure 6D), consistent with acceptable permutation behavior under the present validation conditions. Overall, the results indicate that the volatile profiles of the commercial-yeast control and the composite sourdough CSB treatments were distinguishable at the multivariate level.

**Figure 7:**
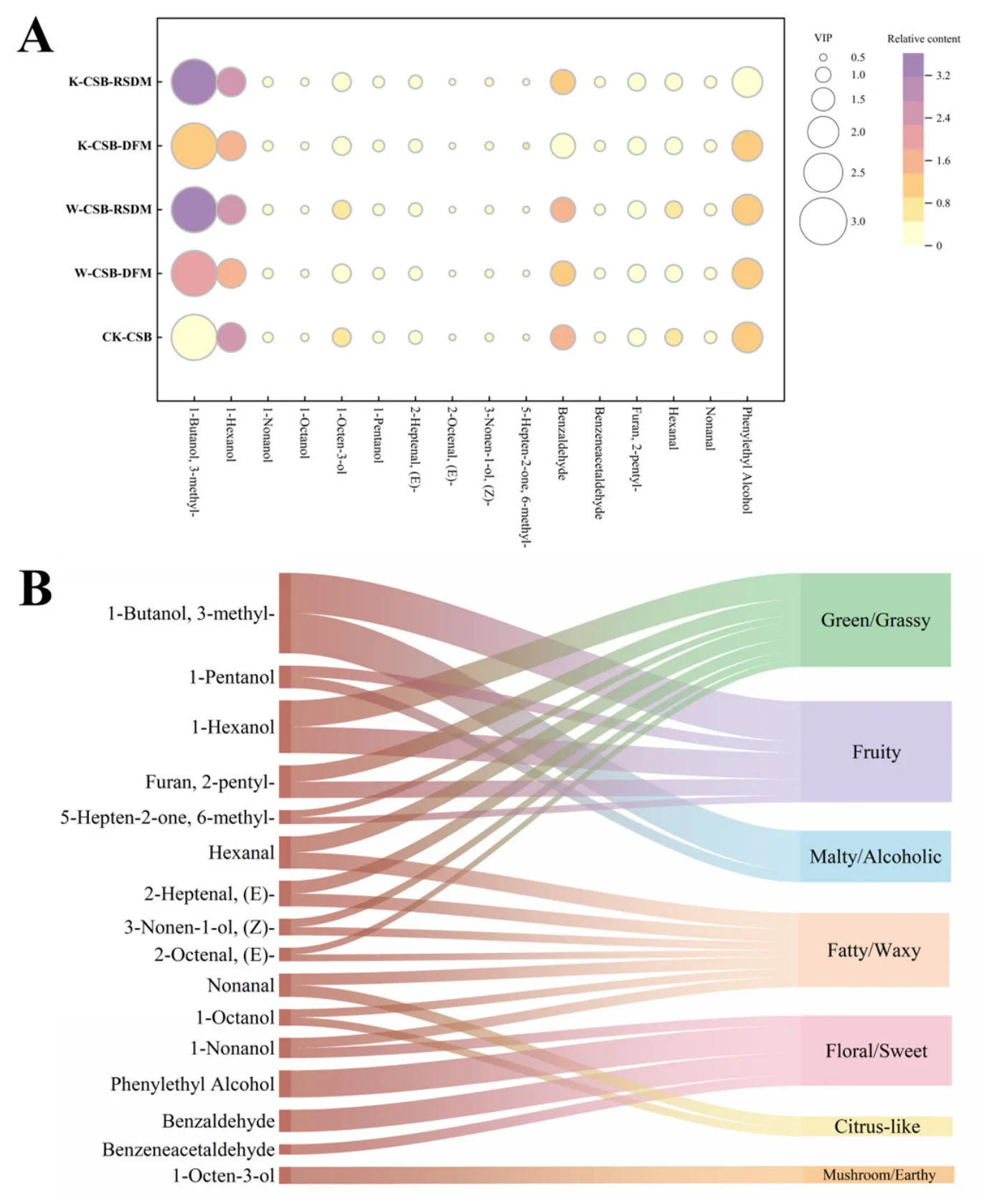
Candidate discriminant volatile organic compounds (VOCs) and their literature-reported odor descriptors in Chinese steamed bread (CSB). (**A**) Bubble plot showing representative candidate discriminant VOCs. (**B**) Sankey diagram linking candidate discriminant VOCs to odor descriptors reported in the literature. The odor descriptors were obtained from published literature and were not experimentally determined in the present study.

The bubble plot summarizes the distribution patterns of representative candidate discriminant VOCs among the five CSB groups (Figure 7A). Among these compounds, 3-methyl-1-butanol was detected in all four composite sourdough CSB samples but not in CK-CSB, indicating an association with composite sourdough treatments under the present conditions. Its mean relative abundances in *W*-CSB-DFM, *W*-CSB-RSDM, *K*-CSB-DFM, and *K*-CSB-RSDM were 2.028, 3.256, 1.122, and 3.634, respectively. Several alcohols and aldehydes also showed treatment-dependent distributions. For example, 1-hexanol was relatively more abundant in *W*-CSB-RSDM and *K*-CSB-RSDM than in the corresponding DFM samples. Benzaldehyde also varied among treatments, with *W*-CSB-RSDM showing a relatively high level among the composite sourdough CSB samples. Ketones and furans likewise contributed to the observed differences. 2-Pentylfuran was relatively more abundant in *W*-CSB-RSDM than in the other composite sourdough CSB groups, whereas lower levels were observed in *K*-CSB-DFM and *K*-CSB-RSDM. Phenylethyl alcohol also exhibited a clear treatment-dependent pattern. It was detected in CK-CSB, *W*-CSB-DFM, *W*-CSB-RSDM, and *K*-CSB-DFM but was not detected in *K*-CSB-RSDM under the present analytical conditions. These results further indicate that the volatile composition of final CSB products was both compound-specific and treatment-dependent. Figure 7B links representative candidate discriminant VOCs to literature-reported odor descriptors, including green/grassy, fruity, malty/alcoholic, fatty/waxy, floral/sweet, citrus-like, and mushroom/earthy descriptors. These literature annotations provide chemical context only and do not establish sensory perception in the present samples.

Overall, composite sourdough treatments were associated with distinct VOC profiles in the final CSB, and the direction and magnitude of the differences depended on the compound and treatment. Among the composite sourdough groups, *W*-CSB-RSDM showed the highest summed internal-standard-normalized VOC signal and relatively high levels of several representative alcohols, aldehydes, and furans. *K*-CSB-RSDM showed a summed internal-standard-normalized VOC signal close to that of CK-CSB but differed in the distribution of individual VOCs.

### 3.8. Specific Volume of CSB

Specific volume differed among the tested formulations (Supplementary Table S6, *p* < 0.05). The commercial-yeast control had a specific volume of 2.44 ± 0.08 mL/g, whereas the four composite sourdough treatments ranged from 2.56 to 2.66 mL/g. *K*-DFM CSB had the highest value (2.66 ± 0.04 mL/g) and was significantly higher than the two RSDM groups, while not differing significantly from *W*-CSB-DFM. Thus, the composite sourdough formulations yielded higher specific volumes than the commercial-yeast control under the tested conditions, although the design does not isolate the causal contributions of LAB, non-*Saccharomyces* yeast, inoculum level, or process history.

### 3.9. Effect of Fermentation Strategy on Volatile Profile Development in CSB

To focus specifically on processing strategy within a fixed starter consortium, DFM and RSDM were compared separately for the *W. anomalus*-*L. plantarum* and *K. marxianus*-*L. plantarum* systems. These pairwise comparisons describe strategy-associated changes within each defined consortium.

#### 3.9.1. *W. anomalus*-Based Composite Sourdough System

**Figure 8:**
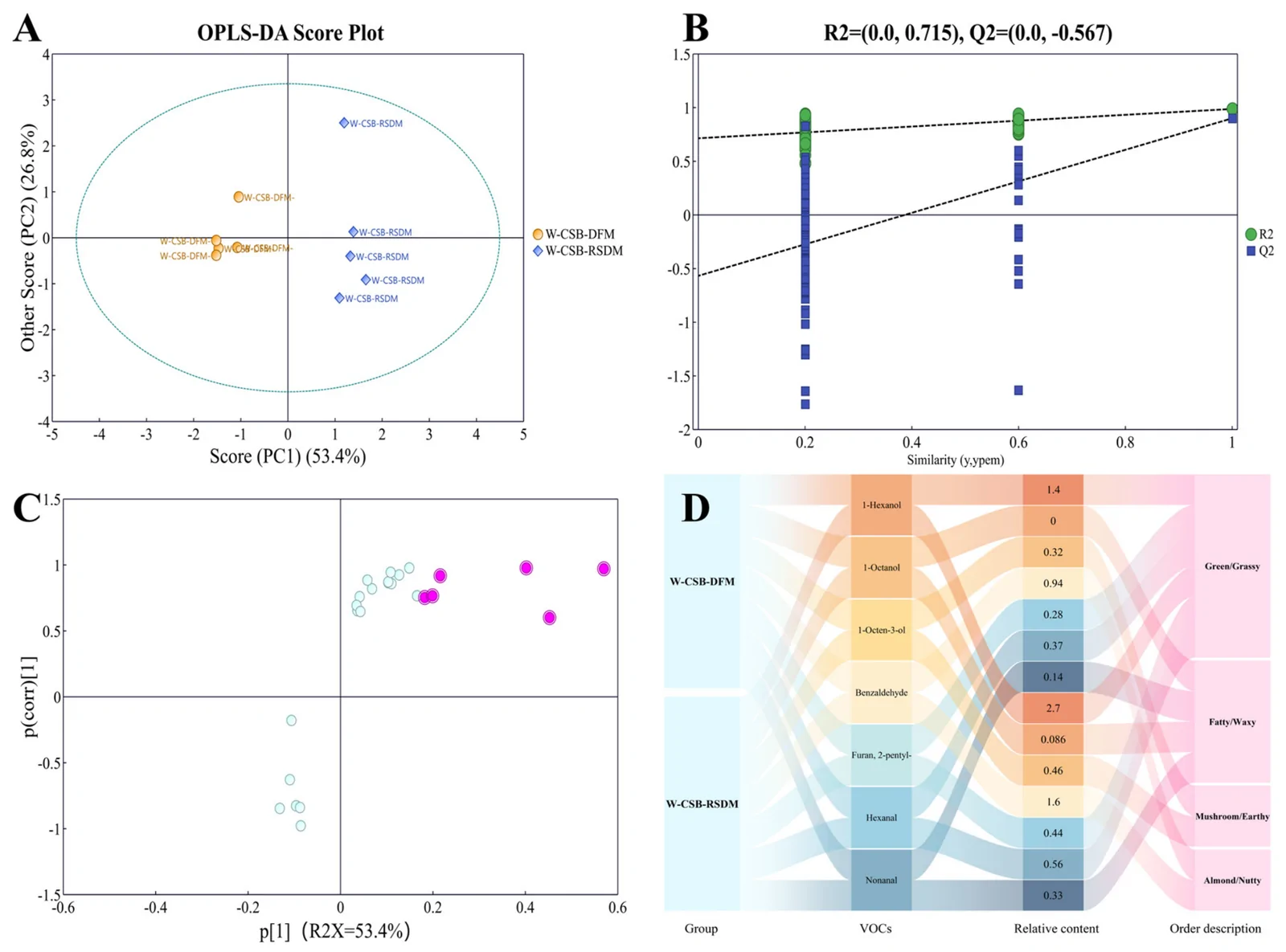
Strategy-associated VOC differences in CSB prepared with the *W. anomalus*-based composite sourdough system. (**A**) Exploratory OPLS-DA score plot comparing DFM and RSDM. (**B**) Permutation test. (**C**) S-plot used to prioritize candidate discriminant VOCs; variables at the extremes show stronger modeled covariance and correlation with the predictive component. Pink dots indicate candidate discriminant VOCs, whereas light blue dots indicate the remaining VOC variables. (**D**) Literature-reported odor descriptors associated with representative candidate discriminant VOCs. The ellipse in panel A indicates the 95% confidence region. In panel B, the dashed lines represent the regression lines of R^2^ and Q^2^ values obtained from the permutation test.

For the *W*-based composite sourdough system (Figure 8), *W*-CSB-DFM and *W*-CSB-RSDM showed treatment-associated separation in the OPLS-DA score space (Figure 8A). The model was evaluated by seven-fold cross-validation and 200 Y-permutation tests. The permutation test yielded a Q^2^ intercept of −0.567 (Figure 8B); however, the R^2^ intercept was relatively high (0.715), indicating less favorable validation behavior than that observed for the other OPLS-DA models in this study. Accordingly, the separation observed in this model was interpreted cautiously and was regarded as supportive evidence of treatment-associated multivariate differences rather than as definitive evidence of class discrimination. The S-plot (Figure 8C) further indicated that the separation between *W*-CSB-DFM and *W*-CSB-RSDM was mainly driven by a limited subset of variables, rather than by uniform changes in all detected VOCs. Most variables were located close to the origin, suggesting limited contribution to group discrimination, whereas several variables with higher covariance and correlation were more closely associated with the separation between the two fermentation strategies. These discriminant variables were mainly related to alcohols, aldehydes, and furan compounds.

Compared with *W*-CSB-DFM, *W*-CSB-RSDM showed higher relative abundances of several representative VOCs. Specifically, 1-hexanol increased from approximately 1.4 to 2.7, 1-octanol increased from undetected or near zero to 0.086, 1-octen-3-ol increased from 0.32 to 0.46, benzaldehyde increased from 0.94 to 1.6, 2-pentylfuran increased from 0.28 to 0.44, hexanal increased from 0.37 to 0.56, and nonanal increased from 0.14 to 0.33. Thus, in the *W*-based composite sourdough system, RSDM was associated with higher relative abundances of several alcohols, aldehydes, and furans in the final CSB. Their commonly reported green/grassy, fatty/waxy, mushroom/earthy, or almond-like descriptors (Figure 8D) are literature annotations only and do not demonstrate perceived sensory differences in the present CSB samples.

#### 3.9.2. *K. marxianus*-Based Composite Sourdough System

**Figure 9:**
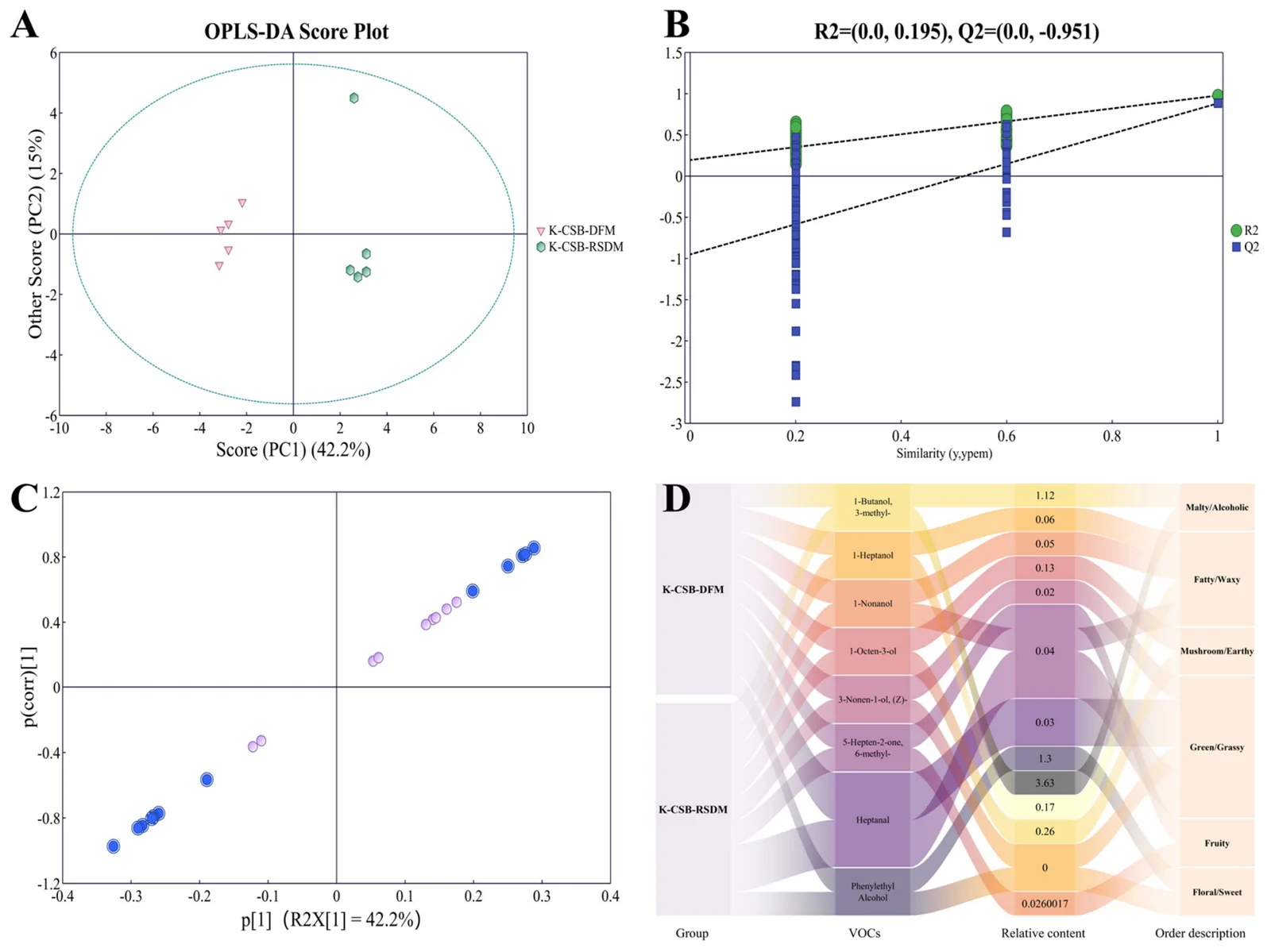
Strategy-associated VOC differences in CSB prepared with the *K. marxianus*-based composite sourdough system. (**A**) Exploratory OPLS-DA score plot comparing DFM and RSDM. (**B**) Permutation test. (**C**) S-plot used to prioritize candidate discriminant VOCs; variables at the extremes show stronger modeled covariance and correlation with the predictive component. Dark blue dots indicate candidate discriminant VOCs, whereas light purple dots indicate the remaining VOC variables. (**D**) Literature-reported odor descriptors associated with representative candidate discriminant VOCs. The ellipse in panel A indicates the 95% confidence region. In panel B, the dashed lines represent the regression lines of R^2^ and Q^2^ values obtained from the permutation test.

For the *K*-based composite sourdough system (Figure 9), *K*-CSB-DFM and *K*-CSB-RSDM also showed clear separation in the OPLS-DA score plot (Figure 9A). The two groups were mainly separated along PC1, which explained 42.2% of the variation, while PC2 explained 15.0%. *K*-CSB-DFM samples were primarily located on the negative side of PC1, whereas *K*-CSB-RSDM samples were distributed on the positive side. This result indicates that fermentation strategy also affected the overall VOC profile of CSB prepared with the K-based composite sourdough. Compared with the *W*-based model, the permutation validation (Figure 9B) of the *K*-based model showed more favorable permutation behavior. The R^2^ intercept was 0.195 and the Q^2^ intercept was −0.951, indicating that the model did not show an obvious overfitting tendency. These results support the use of the model for exploratory examination of fermentation-strategy-associated differences.

The S-plot (Figure 9C) showed that only a subset of detected VOCs contributed strongly to the separation between *K*-CSB-DFM and *K*-CSB-RSDM. Most variables clustered near the origin, whereas several variables located toward the two extremes exhibited relatively high covariance and correlation with the predictive component. After applying the predefined screening criteria of VIP > 1.0 and *p* < 0.05, representative candidate discriminant VOCs included 3-methyl-1-butanol, 1-heptanol, 1-nonanol, 1-octen-3-ol, heptanal, 6-methyl-5-hepten-2-one, and phenylethyl alcohol. The distribution of these compounds indicates that the response of RSDM in the *K*-based system was selective rather than uniform.

At the compound level, 3-methyl-1-butanol showed the most evident increase in *K*-CSB-RSDM compared with *K*-CSB-DFM, increasing from approximately 1.12 to 3.63. This compound is commonly associated with malty/alcoholic descriptors and therefore contributed substantially to the discrimination of *K*-CSB-RSDM from *K*-CSB-DFM. In addition, several aliphatic alcohols and carbonyl compounds, including 1-heptanol, 1-nonanol, 1-octen-3-ol, heptanal, and 6-methyl-5-hepten-2-one, showed strategy-dependent distribution patterns. These compounds were associated with fatty/waxy, green/grassy, mushroom/earthy, and fruity odor descriptors (Figure 9D). Notably, phenylethyl alcohol showed a different pattern from 3-methyl-1-butanol. It was relatively more prominent in *K*-CSB-DFM, whereas it was not detected or was detected only at a very low level in *K*-CSB-RSDM under the present analytical conditions. Because phenylethyl alcohol is commonly associated with floral/sweet descriptors, this result indicates that RSDM did not uniformly increase all potentially aroma-related VOCs in the *K*-based system. Instead, it selectively enhanced compounds such as 3-methyl-1-butanol and some aliphatic alcohols or carbonyl compounds, while reducing the relative abundance or detectability of certain floral/sweet-associated compounds.

#### 3.9.3. Comparison of *W. anomalus*- and *K. marxianus*-Based Systems

Across both defined consortia, RSDM was associated with a different final VOC profile from DFM, but the compounds showing the largest changes were not the same. In the *W*-based system, the principal increases were observed for 1-hexanol, 1-octanol, 1-octen-3-ol, benzaldehyde, 2-pentylfuran, hexanal, and nonanal. In the *K*-based system, the most prominent change was the increase in 3-methyl-1-butanol, accompanied by changes in selected aliphatic alcohols, aldehydes, and ketones, while phenylethyl alcohol decreased or was not detected after RSDM. These observations show joint dependence on process strategy and non-*Saccharomyces* strain identity under the present experimental design. The compounds are therefore best regarded as strategy-associated candidate discriminant VOCs. Any connection to green, fruity, malty, floral, or other perceived sensory attributes remains hypothetical until verified by sensory-directed analytical methods.

## 4. Discussion

The working hypothesis was that defined non-*Saccharomyces* yeast–*L. plantarum* composite sourdoughs would produce stage-, strain-, and process-dependent VOC profiles in CSB. The data support this restricted hypothesis: mature sourdoughs, refrigerated sponge doughs, fully proofed doughs, and final products showed different normalized VOC patterns, and RSDM responses differed between the *W. anomalus*- and *K. marxianus*-based consortia. The following discussion separates these direct observations from mechanistic explanations inferred from established flavor chemistry.

### 4.1. Technological Suitability of the Selected Starter Strains for Composite Sourdough Fermentation

The strain-screening data support the technological suitability of the selected consortium members under the tested conditions without implying that they are globally optimal starters. *L. plantarum* SDgsM3 retained growth under maltose-rich, saline, and acidic conditions and rapidly lowered pH in the flour model. Such traits are relevant because LAB-driven acidification can alter the dough microenvironment, microbial competition, and substrate availability [9,39]. *W. anomalus* CRFSZY16 and *K. marxianus* FYY7 also retained growth under several fermentation-related stresses. Their use is consistent with published evidence that non-*Saccharomyces* bakery yeasts can differ substantially in leavening and volatile-production traits [9,40].

Both yeasts were strongly inhibited by acetic acid in the present assays, emphasizing that organic acid composition can matter independently of bulk pH. The preliminary safety screen detected no hemolysis, yeast phospholipase activity, or LAB gelatinase activity under the tested conditions; however, these assays do not constitute a complete toxicological or genomic safety assessment.

### 4.2. Stage-Dependent Remodeling of Volatile Profiles During CSB Processing

The observed stage-dependent VOC changes should be viewed as the net result of several processes: microbial metabolism during fermentation, endogenous enzymatic reactions, release and retention in the dough matrix, and physicochemical or heat-associated changes during steaming. Because viable fermentation largely precedes steaming, differences that arise during the final heating step cannot be attributed solely to continued microbial metabolism.

In the mature composite sourdoughs and mature refrigerated sponge doughs, alcohols, aldehydes, esters, and ketones were the dominant volatile classes. Compared with the mature composite sourdoughs, the mature refrigerated sponge doughs showed higher summed internal-standard-normalized VOC signals and a higher aldehyde proportion. The additional refrigerated sponge-dough stage therefore provided a longer biochemical transformation window, during which the formation, conversion, release, and retention of individual VOCs could differ from those in the mature sourdough stage. However, the change was selective rather than universal. Some esters decreased or were retained differently depending on the starter system, whereas aldehydes and selected alcohols increased. Therefore, the refrigerated sponge-dough stage should be interpreted as a selective remodeling step rather than a general amplification of all aroma-related compounds. This interpretation is consistent with recent work showing that sourdough fermentation and defined starter combinations can alter texture, flavor, and volatile profiles in cereal-based fermented products [41,42]. In steamed bun or CSB-related systems, mixed fermentation involving LAB, sourdough, yeast, or co-cultures has also been reported to affect product quality, storage characteristics, and volatile composition [13,43]. The present study extends these observations by showing stage-dependent VOC remodeling in defined non-*Saccharomyces* yeast–*L. plantarum* composite sourdough systems during CSB processing.

### 4.3. Strain-Dependent Responses of Non-Saccharomyces Yeast–LAB Composite Sourdoughs to Fermentation Strategy

Within each fixed consortium, DFM and RSDM yielded distinguishable VOC patterns, but the *W*-based and *K*-based systems responded differently. This indicates joint dependence on strain identity and process history under the tested conditions. It does not, however, establish a formal statistical interaction or the independent contribution of each microorganism because yeast-only and LAB-only factorial controls were not included.

In the *W*-based system, RSDM mainly increased the relative abundances of several alcohols, aldehydes, and furans, including 1-hexanol, 1-octanol, 1-octen-3-ol, benzaldehyde, 2-pentylfuran, hexanal, and nonanal. According to odor descriptors reported in the literature, these compounds are commonly associated with green or grassy, fatty or waxy, mushroom or earthy, and almond- or nut-like notes. In contrast, the most prominent response of the *K*-based system to RSDM was a marked increase in the relative abundance of 3-methyl-1-butanol, accompanied by changes in selected aliphatic alcohols, aldehydes, and ketones. Under the present experimental and analytical conditions, the relative abundance of 2-phenylethanol was reduced or the compound was not detected in *K*-CSB-RSDM. These findings demonstrate that RSDM selectively altered the accumulation, release, conversion, or retention of particular VOCs or compound classes depending on the composite starter system.

The contrasting responses of the *W*- and *K*-based systems further support the strain-specific nature of non-*Saccharomyces* yeast functionality in composite sourdough fermentation. Previous reviews and experimental studies have shown that non-*Saccharomyces* yeasts may participate in dough fermentation through carbohydrate utilization, extracellular and intracellular enzyme activities, organic acid and alcohol metabolism, and VOC formation. However, these traits can differ substantially among species and strains and should not be generalized solely on the basis of their classification as non-*Saccharomyces* yeasts [40,44]. Accordingly, the distinct VOC patterns associated with CRFSZY16 and FYY7 are more appropriately interpreted as different net volatile outcomes generated under specific LAB combinations, dough matrices, and fermentation conditions.

Collectively, these results indicate that strain identity and fermentation strategy jointly influenced the final VOC profiles under the present experimental conditions. The same fermentation process may produce different volatile outcomes when combined with different non-*Saccharomyces* yeast–LAB consortia, while the same consortium may show different compound-enrichment patterns under different fermentation regimes. Starter selection for composite sourdough should therefore not be based solely on taxonomic identity or a single fermentation trait. Instead, strain characteristics, target volatile attributes, processing strategy, and the actual dough matrix should be evaluated jointly. From an industrial perspective, RSDM may offer potential for the selective modulation of specific volatile groups in CSB.

### 4.4. Literature-Based Hypotheses for the Formation of Representative Candidate Discriminant VOCs in CSB

**Figure 10:**
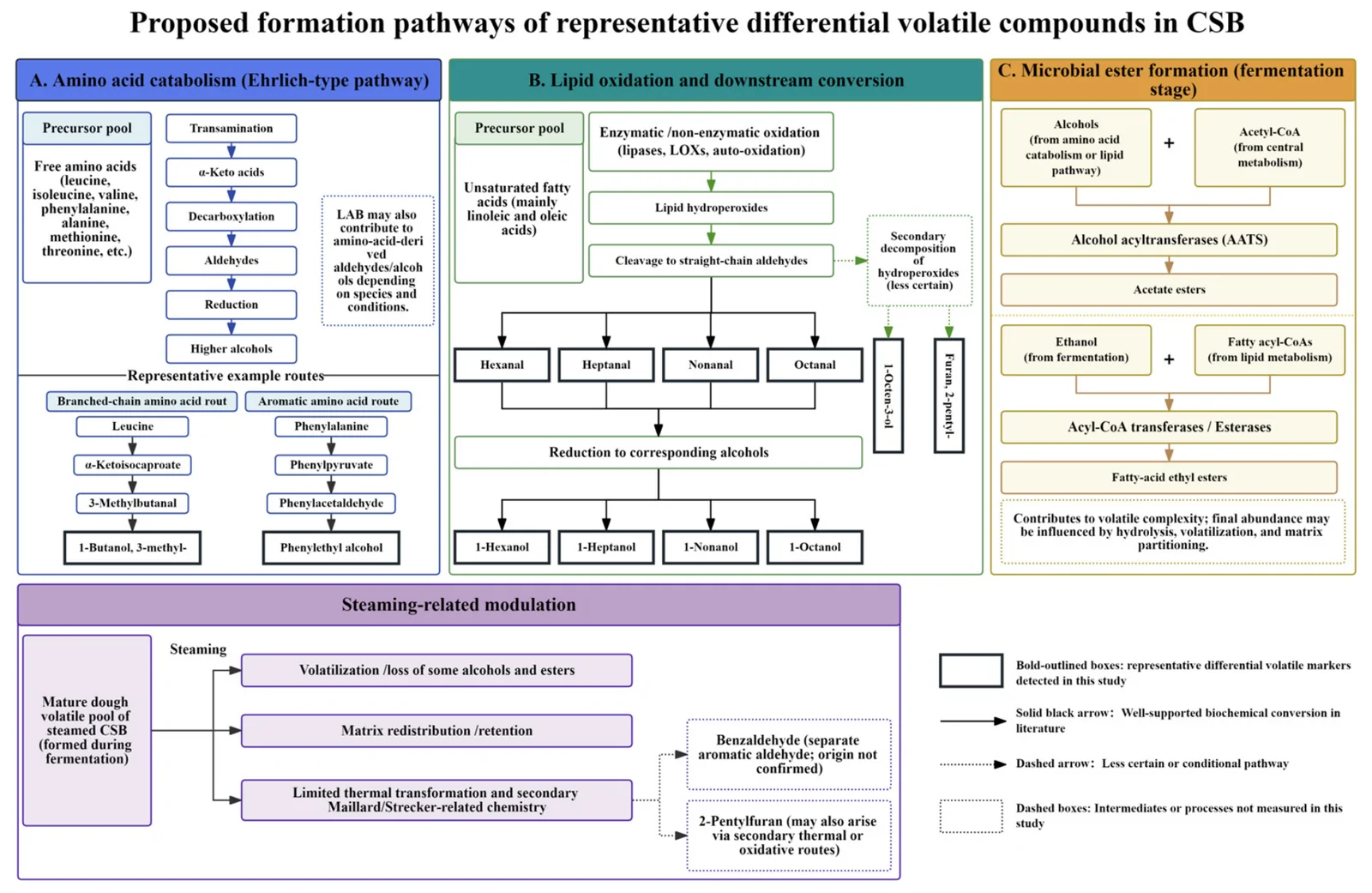
Literature-based hypotheses for the formation and process modulation of representative candidate discriminant VOCs in composite sourdough CSB. The pathways summarize plausible routes and are not experimentally verified metabolic fluxes.

A pathway framework was constructed from the representative candidate discriminant VOCs detected here and established food-flavor chemistry (Figure 10). It is explicitly a hypothesis-generating framework: no isotope tracing, precursor depletion, enzyme assay, transcriptomics, or metabolic-flux experiments were performed, so the proposed arrows should not be interpreted as experimentally demonstrated precursor-product relationships.

One plausible branch involves amino acid catabolism through Ehrlich-type reactions. Amino acids may first be transaminated to their corresponding α-keto acids, which are subsequently decarboxylated to aldehydes and reduced to higher alcohols [45,46]. Leucine can therefore be converted through α-ketoisocaproate and 3-methylbutanal to 3-methyl-1-butanol, whereas phenylalanine can be converted through phenylpyruvate and phenylacetaldehyde to 2-phenylethanol. LAB may also produce amino-acid-derived aldehydes, alcohols, and organic acids, although the product spectrum and conversion efficiency depend strongly on species, strain, substrate availability, and environmental conditions [47]. The comparatively high 3-methyl-1-butanol signal in *K*-CSB-RSDM is compatible with a greater net contribution from branched-chain amino acid-associated chemistry, but the present data cannot determine whether the change arose from formation rate, conversion, release, retention, or steaming loss. In the present study, the *K*-based system showed a particularly notable divergence between these two higher alcohols during RSDM: 3-methyl-1-butanol was markedly enriched in *K*-CSB-RSDM, whereas 2-phenylethanol was reduced to a very low level or was not detected under the same analytical conditions. This contrasting response indicates that amino-acid-derived higher alcohols did not vary uniformly during RSDM and is compatible with differential contributions from branched-chain- and aromatic-amino-acid-associated chemistry. However, the observed abundance patterns cannot establish metabolic rerouting or differential pathway flux. Because precursor amino-acid concentrations, intermediate metabolites, relevant enzyme activities, and metabolic fluxes were not directly measured, the higher 3-methyl-1-butanol signal and lower 2-phenylethanol signal may reflect differences in formation, downstream conversion, release, matrix retention, volatilization, or loss during steaming. These findings should therefore be interpreted as pathway-compatible associations linking the observed VOC profiles with established amino-acid catabolic chemistry rather than as direct evidence of altered metabolic pathways.

A second plausible branch involves unsaturated fatty acid oxidation and downstream carbonyl chemistry. Straight-chain aldehydes and their corresponding alcohols, together with 1-octen-3-ol, 2-pentylfuran, and selected C8 ketones, are chemically compatible with the oxidation of unsaturated fatty acids followed by hydroperoxide cleavage, reduction, cyclization, or other secondary reactions. In cereal systems, hexanal is commonly associated with linoleic acid oxidation, whereas nonanal is often linked to oleic acid oxidation; however, these assignments are not exclusive because multiple hydroperoxide and secondary reaction routes can converge on the same volatile product. Linoleic acid-derived chemistry can also contribute to 1-octen-3-ol and 2-pentylfuran [5,48,49]. The comparatively high abundances of 1-hexanol, hexanal, nonanal, and 2-pentylfuran in *W*-CSB-RSDM are consistent with a stronger net expression of lipid-derived chemistry in the *W*-based system. In addition, steaming itself can favor the appearance of benzaldehyde and 2-pentylfuran while promoting the loss of alcohols and esters [50], so final headspace abundance reflects both formation and process-dependent retention or loss.

Ester abundance can likewise reflect several competing processes. Yeast acetate esters are generally formed through the condensation of alcohols with acetyl-CoA catalyzed by alcohol acetyltransferases, whereas the formation of fatty acid ethyl esters can involve additional acyl-CoA transferases or esterases [51,52,53]. Sourdough fermentation can provide the alcohol and acid/acyl-donor pools needed for ester formation [5,53]. Higher yeast level and longer fermentation times in CSB have also been associated with greater ester generation, whereas steaming promotes ester loss [50]. Consequently, acetate esters and fatty acid ethyl esters detected in the present samples may reflect fermentation-stage precursor availability and enzymatic ester synthesis, but their final headspace abundance will additionally depend on hydrolysis, volatilization, and matrix partitioning.

In contrast to fermentation, the steaming step should be interpreted primarily as a thermal and physicochemical processing stage rather than as a continuation of microbial fermentation. Heating can reshape the final headspace VOC profile through volatilization, matrix redistribution and partitioning, differential retention, and thermally promoted secondary transformations of compounds or precursors accumulated during fermentation. Previous CSB research demonstrated that 3-methyl-1-butanol increased during fermentation but decreased during steaming, whereas prolonged steaming promoted the appearance of benzaldehyde and 2-pentylfuran and enhanced the loss of alcohols and esters [50]. These observations indicate that changes detected after steaming cannot be attributed solely to microbial biosynthesis occurring during the preceding fermentation stage. The formation or enrichment of selected aldehydes and furans during heating may also be compatible with secondary reactions involving lipid-derived intermediates. In cereal systems, straight-chain aldehydes and 2-pentylfuran have been associated with unsaturated fatty acid oxidation and subsequent decomposition or conversion of lipid hydroperoxides [48,49]. Thus, the increased abundance of selected carbonyl compounds or furans after steaming may reflect heat-facilitated transformation of pre-existing lipid-derived precursors or intermediates in addition to differences in volatilization and matrix retention. However, because the present study did not directly measure lipid hydroperoxides, thermal-reaction intermediates, or reaction kinetics, the specific chemical routes responsible for these changes cannot be assigned conclusively. Moreover, because CSB is processed under moist steaming conditions rather than the higher-temperature, lower-moisture conditions characteristic of baking, extensive Maillard-derived aroma formation should not be assumed. Accordingly, the final CSB volatile profile is more appropriately interpreted as the integrated outcome of microbial precursor and VOC formation during fermentation followed by selective thermal formation, transformation, loss, redistribution, and retention during steaming.

**Figure 11:**
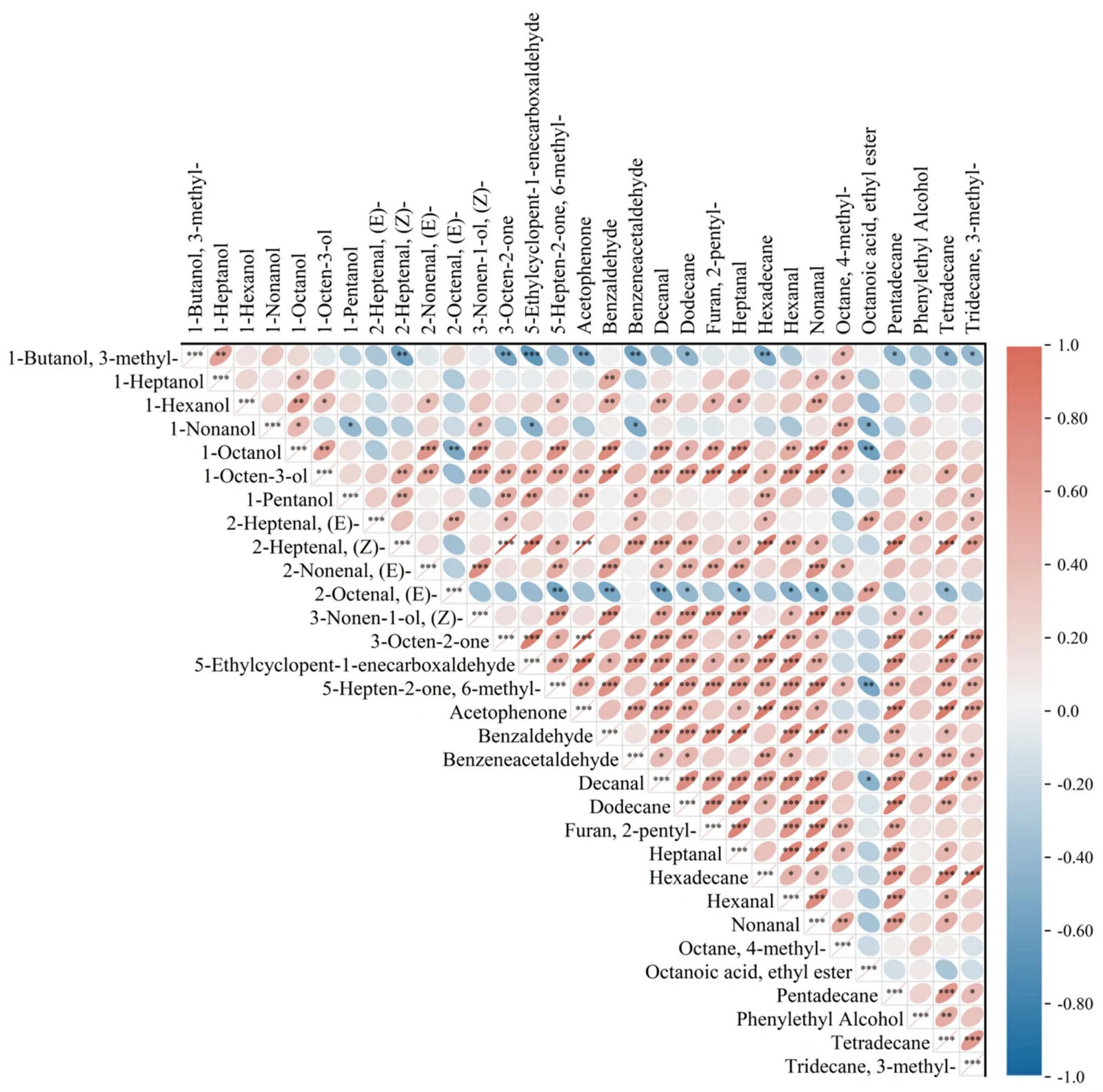
Pearson correlation heatmap of VOCs in composite sourdough CSB. Asterisks indicate statistical significance (* *p* < 0.05, ** *p* < 0.01, and *** *p* < 0.001). Correlations describe coordinated variation among compounds and do not establish causal metabolic relationships.

Correlation analysis provided complementary information on co-variation patterns relevant to the proposed VOC formation pathways (Figure 11). Significant correlations were observed among several alcohols, aldehydes, ketones, and furans. In particular, the positive correlations among straight-chain aldehydes, their corresponding straight-chain alcohols, 1-octen-3-ol, and 2-pentylfuran were compatible with shared contributions from unsaturated fatty acid oxidation, lipid hydroperoxide cleavage, aldehyde reduction, and secondary oxidative degradation. The co-variation between phenylacetaldehyde and 2-phenylethanol was also compatible with phenylalanine catabolism through an Ehrlich-type pathway. However, correlation reflects coordinated variation across treatments and cannot establish direct precursor–product relationships or metabolic fluxes.

Overall, the refrigerated sponge-dough method (RSDM) modulated the detectable VOC profile in a starter-system- and dough-matrix-dependent manner. In the *K*-based system, the higher abundance of 3-methyl-1-butanol was compatible with a greater net contribution from branched-chain amino acid-associated higher-alcohol chemistry. In the *W*-based system, the higher levels of hexanal, nonanal, 1-hexanol, and 2-pentylfuran were more consistent with a greater contribution from lipid-derived chemistry. These mechanistic interpretations remain hypotheses because the measured signals were also shaped by matrix effects and by transformation, volatilization, and retention during steaming.

### 4.5. Relationship Between Composite Sourdough Fermentation and CSB Specific Volume

In addition to modifying VOC profiles, the composite sourdough treatments were associated with higher specific volumes than the commercial-yeast control under the tested formulations. This result suggests that the selected composite sourdough systems were compatible with gas expansion and structure development under the present formulation and processing conditions. The higher specific volume could reflect microbial gas production, moderate acidification, and modification of dough structure. However, the relationship between sourdough fermentation and volume should not be generalized as universally positive. Excessive acidification or unsuitable starter combinations may weaken gluten structure, reduce gas retention, and negatively affect product volume. Recent studies on steamed bun, CSB, and cereal-based sourdough systems also indicate that LAB, yeast, sourdough, and co-culture fermentation can affect product quality, texture, storage behavior, and sensory properties, but the direction and magnitude of these effects depend on the starter and processing conditions [13,41,43]. Future factorial controls matched for yeast cell number and fermentation history would be needed to partition these effects.

### 4.6. Limitations and Future Perspectives

The VOC data were obtained using HS-SPME-GC-TQ-MS and expressed as internal-standard-normalized relative abundances, which reflect relative differences in detectable volatile levels rather than absolute concentrations. The present study did not include sensory evaluation, GC-olfactometry, or odor-activity-value analysis. Accordingly, literature-derived descriptors such as green, fruity, floral, malty, or mushroom-like should not be interpreted as demonstrated sensory attributes of the present samples. Future studies should combine targeted quantification, odor thresholds, odor activity values, GC-olfactometry, aroma recombination/omission experiments, trained descriptive sensory evaluation, and consumer acceptance or liking tests to determine which VOC differences are perceptible and relevant to consumer preference [54,55]. Moreover, the proposed formation pathways were inferred from the detected candidate discriminant VOCs and established flavor chemistry and should be further evaluated through precursor profiling, metabolomics, and enzyme- or gene-level analyses. Despite these limitations, the present study provides a controlled basis for future sensory-directed and mechanistic validation of strain- and process-dependent VOC differences in CSB.

## 5. Conclusions

Under the tested formulations and analytical conditions, defined *W. anomalus*–*L. plantarum* and *K. marxianus*–*L. plantarum* composite sourdoughs produced distinct stage-, strain-, and process-dependent VOC profiles in Chinese steamed bread. Compared with direct fermentation, the refrigerated sponge-dough method selectively altered representative alcohols, aldehydes, esters, furans, and ketones, rather than uniformly increasing all VOCs. The *W*- and *K*-based composite sourdough systems responded differently to RSDM, underscoring the need to evaluate non-*Saccharomyces* yeasts at the strain level for flavor-oriented CSB production. Composite sourdough treatments also yielded higher specific volumes than the commercial-yeast control under the tested conditions. These findings provide a practical basis for strain- and process-specific composite sourdough design but do not resolve the individual contribution of each microorganism, establish a universal yeast-LAB synergistic effect, or demonstrate sensory superiority. Sensory-directed and mechanistic studies are therefore required before the observed VOC differences can be translated into consumer-relevant flavor claims.

## Data Availability

The original contributions presented in this study are included in the article/Supplementary Materials. Further inquiries can be directed to the corresponding author.

## Supplementary Materials

The following supporting information can be downloaded at: https://www.mdpi.com/article/10.3390/foods15173064/s1. Figure S1. Experimental design and sampling scheme; Table S1. Microbial strains used in this study; Table S2. Formulation for Chinese Steamed Bread (CSB) Preparation; Table S3. Volatile Profiles of Mature Composite Sourdoughs and Refrigerated Sponge Dough; Table S4. Volatile Profiles of Fully Proofed Main Doughs Prepared with Different Starters; Table S5. Volatile Profiles of CSB Prepared with Composite Sourdoughs; Table S6. Specific volume of CSB prepared with different starter systems.

## Abbreviations

The following abbreviations are used in this manuscript:

*S. cerevisiae*	*Saccharomyces cerevisiae*
*W. anomalus* (*W*)	*Wickerhamomyces anomalus*
*K. marxianus* (*K*)	*Kluyveromyces marxianus*
*L. plantarum* (*L*)	*Lactiplantibacillus plantarum*
LAB	lactic acid bacteria
YPD	yeast extract–peptone–dextrose broth/agar
MRS	de Man, Rogosa and Sharpe broth/agar
YNB	yeast nitrogen base
OD600	optical density at 600 nm
CBFM	cereal-based fermentation medium
HS-SPME-GC-TQ-MS	headspace solid-phase microextraction coupled with gas chromatography-triple quadrupole mass spectrometry
RI	retention index
VOCs	volatile organic compounds
PCA	principal component analysis
OPLS-DA	orthogonal partial least squares-discriminant analysis
VIP	variable importance in projection
MIC	minimum inhibitory concentration
AMP	ampicillin
Cm	chloramphenicol
Gen	gentamicin
TET	tetracycline
E	erythromycin
S	streptomycin
DY	dough yield
TTA	total titratable acidity
MSD	mature sourdough
MRSD	mature refrigerated sponge dough
*W*-MSD	MSD co-fermented with *W. anomalus* and *L. plantarum*
*K*-MSD	MSD co-fermented with *K. marxianus* and *L. plantarum*
*W*-MRSD	MRSD co-fermented with *W. anomalus* and *L. plantarum*
*K*-MRSD	MRSD co-fermented with *K. marxianus* and *L. plantarum*
MD	main dough (fully proofed final dough)
DFM	direct fermentation method
RSDM	refrigerated sponge-dough method
CK-MD	Commercial-yeast control main dough
*W*-MD-DFM	*W*-type composite sourdough main dough prepared by DFM
*W*-MD-RSDM	*W*-type composite sourdough main dough prepared by RSDM
*K*-MD-DFM	K-type composite sourdough main dough prepared by DFM
*K*-MD-RSDM	K-type composite sourdough main dough prepared by RSDM
CSB	Chinese steamed bread
CK-CSB	commercial-yeast control Chinese steamed bread
*W*-CSB-DFM	*W*-type composite-sourdough Chinese steamed bread prepared by DFM
*W*-CSB-RSDM	*W*-type composite-sourdough Chinese steamed bread prepared by RSDM
*K*-CSB-DFM	*K*-type composite-sourdough Chinese steamed bread prepared by DFM
*K*-CSB-RSDM	*K*-type composite-sourdough Chinese steamed bread prepared by RSDM
